# Supplementary material for: Evolutionary Toxicogenomics of the Striped Killifish (Fundulus majalis) in the New Bedford Harbor (Massachusetts, USA)
Source: Int J Mol Sci. 2019 Mar 5;20(5):1129. doi: 10.3390/ijms20051129 (PMC6429206; doi:10.3390/ijms20051129)
Supplement: Supplementary file 1 [file ijms-20-01129-s001.pdf]

SUPPLEMENTARY FIGURES AND TABLES TO RUGGERI ET AL. (XXXX)

Figure S1. STRUCTURE_Simulations outcomes based on the use of non-neutral loci (outliers + loci under linkage disequilibrium) [Figure S1 a-b], based on exclusively neutral loci [Figure S1 c-d] and based on the whole set of 4128 SNPs [Figure S1 e-f]. Plots a-c-d refers to the mean Logarithmic Probability values for any simulated K clusters. Plots b-d-f represent the best K based on the Evanno method.

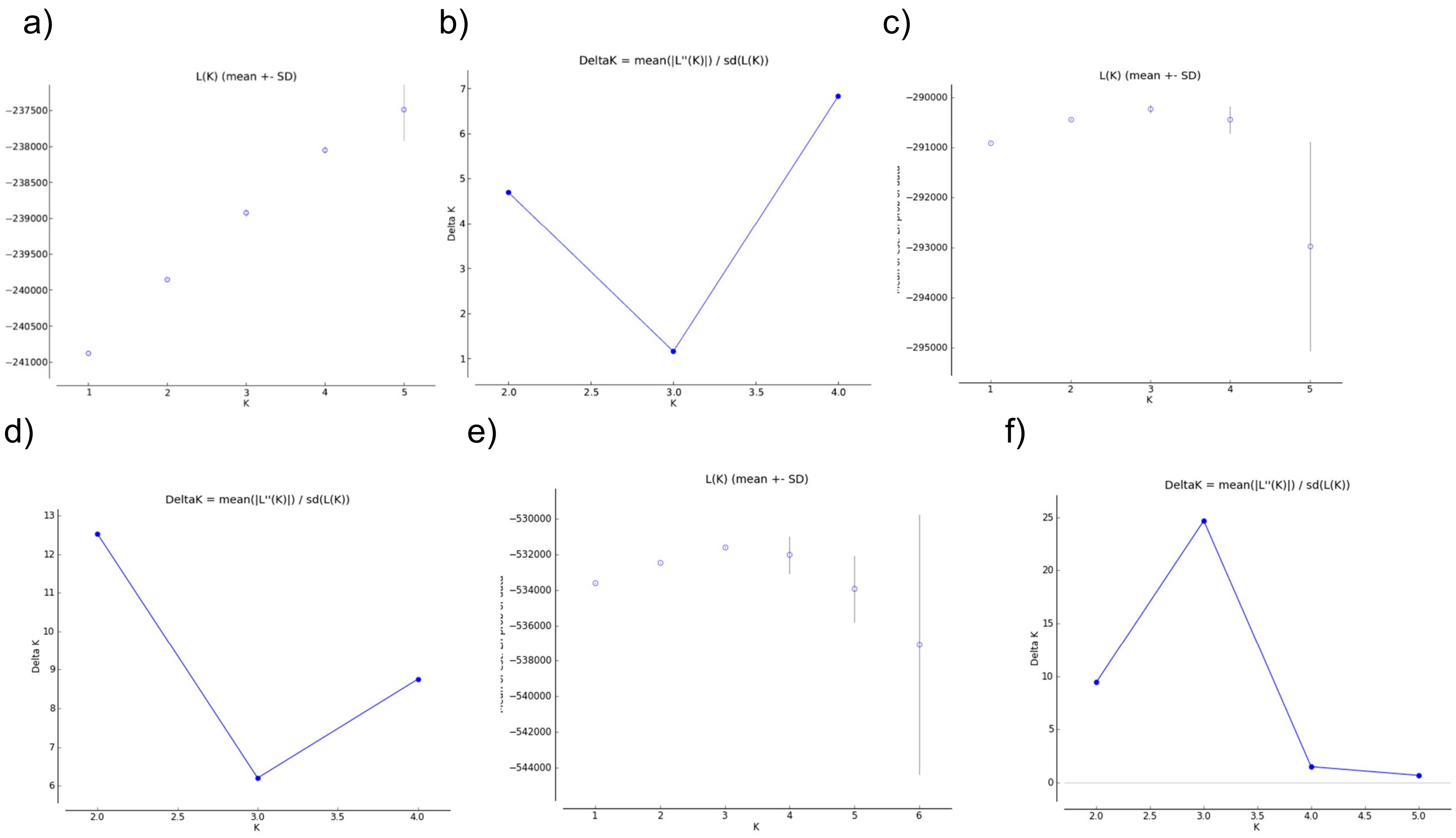

Figure S2. Annotations percentages by Taxonomical groups of living beings (upper pie chart) and Fish Orders (lower pie chart).

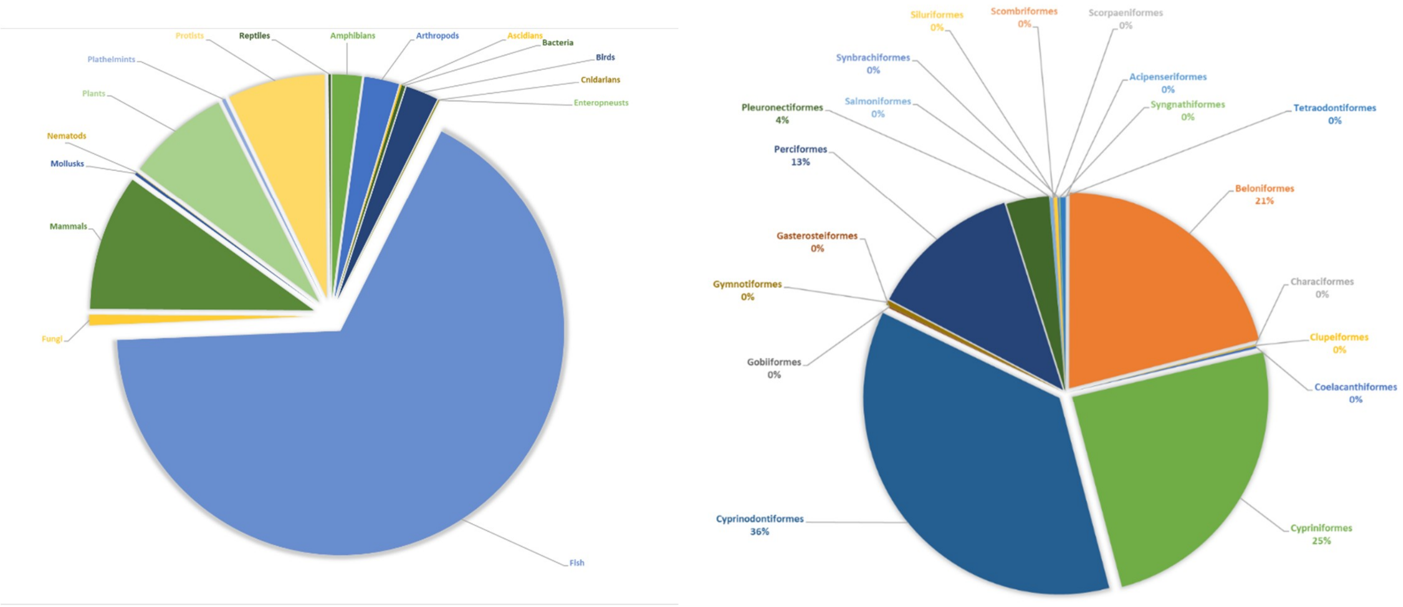

Table S1. AMOVA results.

Source of variation	Sum of Squares	Variance components	Percentage of variation (%)	
**Among groups (FCT)**	1,771.87	1.67	0.23 (***)	FCT : 0.0032
**Among populations within groups (FSC)**	761.41	0.72	0.10 (ns)	FSC : 0.0010
**Within populations (FST)**	175,285.28	736.15	99.67 (***)	FST : 0.0023

*** P-value < 0.001. ns = no significances; Group 1 = PIL+FAH, Group 2 = HST, Group 3 = MAT.

Table S2. First generation migrants. The table above shows the percentage of first-generation migrants among sampling sites (first column) and their hypothetical site of origin based on the multilocus profile of each individual.

	**SITE OF ORIGIN**			
	**PIL**	**FAH**	**HST**	**MAT**
**PIL**	11.43	25.71	45.71	17.14
**FAH**	29.03	12.9	35.48	22.58
**HSS**	38.89	27.78	16.67	16.67
**MAT**	32.35	26.47	32.35	8.82

Table S3. List of annotated hits and their corresponding human Uniprot accession numbers used for the enrichment analysis in DAVID 6.7.

N	ncbi accession number	Method	Locus name	Hits	UNIPROT (human*)
1	XM_022195682.1	L	**S9991_599090**	Acanthochromis polyacanthus multidrug resistance-associated protein 4-like (LOC110952229), mRNA	O15439
					A0A2R8Y6V8
					A0A2R8YDC1
2	XM_022196756.1	B	**S1073_18989**	Acanthochromis polyacanthus PHD finger protein 20-like (LOC110952979), transcript variant X1, mRNA	Q9BVI0
	XM_022196757.1			Acanthochromis polyacanthus PHD finger protein 20-like (LOC110952979), transcript variant X2, mRNA	
3	XM_022193155.1	L + B	**S9899_1841030**	Acanthochromis polyacanthus syntaxin-2-like (LOC110950537), transcript variant X1, mRNA	J3KNU7
	XM_022193156.1			Acanthochromis polyacanthus syntaxin-2-like (LOC110950537), transcript variant X2, mRNA	A0A348AY69
	XM_022193157.1			Acanthochromis polyacanthus syntaxin-2-like (LOC110950537), transcript variant X3, mRNA	
4	XM_019794196.1	H	**S217_136844**	Ailuropoda melanoleuca DEF6, guanine nucleotide exchange factor (DEF6), transcript variant X1, mRNA	Q9H4E7
					Q01524
					B3KSI1
					D2DEF6
					H0Y7V7
5	XM_023266513.1	L	**S124_317291**	Amphiprion ocellaris Rho guanine nucleotide exchange factor 10 like (arhgef10l), transcript variant X1, mRNA	Q9HCE6
	XM_023266514.1			Amphiprion ocellaris Rho guanine nucleotide exchange factor 10 like (arhgef10l), transcript variant X2, mRNA	Q5VXI4
	XM_023266516.1			Amphiprion ocellaris Rho guanine nucleotide exchange factor 10 like (arhgef10l), transcript variant X3, mRNA	A0A2X0U2F8
	XM_023266517.1			Amphiprion ocellaris Rho guanine nucleotide exchange factor 10 like (arhgef10l), transcript variant X4, mRNA	A0A384DVK7
	XM_023266518.1			Amphiprion ocellaris Rho guanine nucleotide exchange factor 10 like (arhgef10l), transcript variant X5, mRNA	
	XM_023266519.1			Amphiprion ocellaris Rho guanine nucleotide exchange factor 10 like (arhgef10l), transcript variant X6, mRNA	
6	XM_023282727.1	L + B	**S9899_1841030**	Amphiprion ocellaris syntaxin-2-like (LOC111576815), transcript variant X1, mRNA	J3KNU7
	XM_023282735.1			Amphiprion ocellaris syntaxin-2-like (LOC111576815), transcript variant X2, mRNA	A0A348AY69
7	XM_016993454.1	L	**S1_2422231**	Anolis carolinensis synaptonemal complex protein 2 (LOC103278796), transcript variant X1, mRNA	Q9BX26

	XM_016993456.1			Anolis carolinensis synaptonemal complex protein 2 (LOC103278796), transcript variant X2, mRNA	
	XM_008110239.2			Anolis carolinensis synaptonemal complex protein 2 (LOC103278796), transcript variant X3, mRNA	
8	KC161997.1	B	**S8943_473**	Anoplopoma fimbria follicle-stimulating hormone receptor mRNA, complete cds	C9JDA1
					F8WBM4
					B6UV79
					A0A1S0QI99
					B4DXB5
					T2HV29
					P23945
					A0A1D5RMN4
9	XM_012472993.1	B	**S3317_962**	Aotus nancymaae jade family PHD finger 1 (JADE1), transcript variant X1, mRNA	Q6IE81
	XM_012472994.1			Aotus nancymaae jade family PHD finger 1 (JADE1), transcript variant X2, mRNA	D6RFK0
	XM_012472995.1			Aotus nancymaae jade family PHD finger 1 (JADE1), transcript variant X3, mRNA	D6RGE7
	XM_012472996.1			Aotus nancymaae jade family PHD finger 1 (JADE1), transcript variant X4, mRNA	D6RC05
					D6RCS1
					D6RBB3
					D6RAS5
					D6RE74
10	XM_009283779.2	L	**S9853_2824879**	Aptenodytes forsteri Yip1 domain family member 4 (YIPF4), transcript variant X1, mRNA	Q9BSR8
					H7C0D5
					H7C3X2
11	XM_011580237.1	L	**S9853_2824879**	Aquila chrysaetos canadensis ubiquitin specific peptidase 9, X-linked (USP9X), transcript variant X1, mRNA	Q93008
	XM_011580238.1			Aquila chrysaetos canadensis ubiquitin specific peptidase 9, X-linked (USP9X), transcript variant X2, mRNA	
12	XM_026145840.1	B	**S8943_473**	Astatotilapia calliptera NME/NM23 family member 9 (nme9), transcript variant X1, mRNA	Q86XW9
	XM_026145841.1			Astatotilapia calliptera NME/NM23 family member 9 (nme9), transcript variant X2, mRNA	Q3KNW3
	XM_026145842.1			Astatotilapia calliptera NME/NM23 family member 9 (nme9), transcript variant X3, mRNA	C9JXP5
	XM_026145843.1			Astatotilapia calliptera NME/NM23 family member 9 (nme9), transcript variant X4, mRNA	H7C5P8
	XM_026145844.1			Astatotilapia calliptera NME/NM23 family member 9 (nme9), transcript variant X5, mRNA	F8WDB2

					C9JPM2
13	XM_026146628.1	B	**S561_8990**	Astatotilapia calliptera potassium voltage-gated channel subfamily A member 1-like (LOC113008816), transcript variant X1, mRNA	Q09470
	XM_026146629.1			Astatotilapia calliptera potassium voltage-gated channel subfamily A member 1-like (LOC113008816), transcript variant X2, mRNA	
14	XM_026147420.1	L	**S533_118016**	Astatotilapia calliptera putative nuclease HARBI1 (LOC113009226), mRNA	E9PQI1
	XM_026166005.1			Astatotilapia calliptera putative nuclease HARBI1 (LOC113021367), mRNA	E9PK24
	XM_026166006.1			Astatotilapia calliptera putative nuclease HARBI1 (LOC113021368), mRNA	Q96MB7
	XM_026172209.1			Astatotilapia calliptera putative nuclease HARBI1 (LOC113024845), mRNA	
	XM_026172212.1			Astatotilapia calliptera putative nuclease HARBI1 (LOC113024847), mRNA	
	XM_026172217.1			Astatotilapia calliptera putative nuclease HARBI1 (LOC113024914), mRNA	
	XM_026180011.1			Astatotilapia calliptera putative nuclease HARBI1 (LOC113029242), mRNA	
15	XM_007238721.3	B	**S9888_703558**	Astyanax mexicanus voltage-dependent L-type calcium channel subunit beta-2 (LOC103035300), transcript variant X1, mRNA	Q08289
	XM_007238723.3			Astyanax mexicanus voltage-dependent L-type calcium channel subunit beta-2 (LOC103035300), transcript variant X2, mRNA	
	XM_007238724.3			Astyanax mexicanus voltage-dependent L-type calcium channel subunit beta-2 (LOC103035300), transcript variant X3, mRNA	
	XM_007238725.3			Astyanax mexicanus voltage-dependent L-type calcium channel subunit beta-2 (LOC103035300), transcript variant X4, mRNA	
16	XM_026861766.1	B	**S8943_473**	Athene cunicularia kinase suppressor of ras 1 (KSR1), transcript variant X1, mRNA	Q8IVT5
	XM_026861767.1			Athene cunicularia kinase suppressor of ras 1 (KSR1), transcript variant X2, mRNA	
	XM_026861768.1			Athene cunicularia kinase suppressor of ras 1 (KSR1), transcript variant X3, mRNA	
	XM_026861770.1			Athene cunicularia kinase suppressor of ras 1 (KSR1), transcript variant X5, mRNA	
	XM_026861771.1			Athene cunicularia kinase suppressor of ras 1 (KSR1), transcript variant X6, mRNA	
17	XM_026857421.1	L	**S885_89904**	Athene cunicularia leucine rich repeat neuronal 1 (LRRN1), mRNA	Q6UXK5
18	XM_014002131.1	L	**S124_317291**	Austrofundulus limnaeus ArfGAP with coiled-coil, ankyrin repeat and PH domains 3 (acap3), transcript variant X4, mRNA	Q96P50
					Q8WTZ1
					Q8N2W2
					F8W850
19	XM_014028472.1	B + H	**S368_129091**	Austrofundulus limnaeus exostosin-1-like (LOC106532427), partial mRNA	D7RTA7
	XM_014028472.1			Austrofundulus limnaeus exostosin-1-like (LOC106532427), partial mRNA	H7C1H6
					S5Y321
					V5QSK8

					T2FFJ4
					F8WF54
20	XM_014008025.1	L	**S2711_3408**	Austrofundulus limnaeus MAGUK p55 subfamily member 4-like (LOC106517273), mRNA	A0A087WUS1
					E9PG92
					E7ET46
					E7EUL8
					R4GN59
					F6Q0Y6
					A2RU80
					B7ZM19
21	XM_014003938.1	B + H	**S368_129091**	Austrofundulus limnaeus protein orai-2-like (LOC106514602), mRNA	Q96SN7
					C9J2H9
					C9JQR7
					C9JUY6
22	XM_014026680.1	B	**S771_52821**	Austrofundulus limnaeus suppression of tumorigenicity 5 (st5), transcript variant X1, mRNA	P78524
	XM_014026687.1			Austrofundulus limnaeus suppression of tumorigenicity 5 (st5), transcript variant X2, mRNA	B4DDL8
					E9PLH5
					H0YE59
					E9PKE0
23	XM_014023439.1	L + B	**S9899_1841030**	Austrofundulus limnaeus syntaxin 2 (stx2), mRNA	J3KNU7
					A0A348AY69
24	XM_014015995.1	L + H	**S42_249281**	Austrofundulus limnaeus thyrotropin-releasing hormone (trh), mRNA	P20396
25	AB106676.1	L	**S178_234532**	Avicennia marina EF1-A mRNA for elongation factor 1A, complete cds	P68104
					Q6IPS9
26	KX758048.1	L	**S178_234532**	Babesia microti Trx1 mRNA, complete cds	P32888
					P52348
27	XM_019049716.1	L	**S1100_29850**	Bemisia tabaci regulator of nonsense transcripts 2 (LOC109035898), mRNA	Q9HAU5
28	XM_010850203.1	H	**S9889_2454843**	Bison bison bison ring finger protein 207 (RNF207), mRNA	Q6ZRF8

29	KM102660.1	L	**S9929_1038014**	Boleophthalmus pectinirostris heat shock protein 70 (hsp70) mRNA, complete cds	Q9NZL4
					P0DMV8
					P0DMV9
30	NM_001252670.1	L	**S1100_29850**	Bombyx mori ubiquitin-conjugating enzyme E2 J1-like (LOC100852386), mRNA	Q9Y385
31	BC153292.1	L	**S9929_1038014**	Bos taurus cytoplasmic polyadenylation element binding protein 4, mRNA (cDNA clone MGC:159945 IMAGE:8466970), complete cds	B7ZLQ8
					E5RFP2
					E5RJM0
					H0YBG1
					D3DQM9
					E1CJT3
					Q17RY0
32	NM_001098086.1	L	**S9929_1038014**	Bos taurus eukaryotic translation initiation factor 2 alpha kinase 3 (EIF2AK3), mRNA	Q9NZJ5
					E7ER02
33	NM_001083512.1	L	**S9929_1038014**	Bos taurus KH RNA binding domain containing, signal transduction associated 3 (KHDRBS3), mRNA	C9J185
					E5RJZ9
					H0YAQ1
					O75525
34	NM_001080730.2	L	**S9929_1038014**	Bos taurus mitochondrial ribosomal protein L39 (MRPL39), mRNA	Q9NYK5
	BC122667.1			Bos taurus mitochondrial ribosomal protein L39, mRNA (cDNA clone MGC:142617 IMAGE:8253321), complete cds	C9JG87
35	BC123798.1	L	**S29_324875**	Bos taurus sarcoglycan, alpha (50kDa dystrophin-associated glycoprotein), mRNA (cDNA clone MGC:143412 IMAGE:8286561), complete cds	Q16586
					A0A0S2Z4P8
36	NM_001081531.2	L	**S29_324875**	Bos taurus small integral membrane protein 8 (SMIM8), mRNA	Q96KF7
					V9GYF9
					V9GYL2
37	XM_025857278.1	L	**S897_124935**	Callorhinus ursinus CRK like proto-oncogene, adaptor protein (CRKL), mRNA	P46108
38	NM_001003140.1	L	**S9929_1038014**	Canis lupus familiaris tight junction protein 1 (TJP1), mRNA	G3V1L9
					Q07157
39	XM_025457624.1	B	**S3317_962**	Canis lupus dingo ELAV like RNA binding protein 3 (ELAVL3), transcript variant X1, mRNA	Q14576

	XM_025457626.1			Canis lupus dingo ELAV like RNA binding protein 3 (ELAVL3), transcript variant X2, mRNA	Q96J71
	XM_025457627.1			Canis lupus dingo ELAV like RNA binding protein 3 (ELAVL3), transcript variant X3, mRNA	K7EPB5
40	XM_005632851.3	B	**S3317_962**	Canis lupus familiaris ELAV like RNA binding protein 3 (ELAVL3), transcript variant X1, mRNA	Q9H024
	XM_022407022.1			Canis lupus familiaris ELAV like RNA binding protein 3 (ELAVL3), transcript variant X2, mRNA	L8E8Z0
	XM_005632852.3			Canis lupus familiaris ELAV like RNA binding protein 3 (ELAVL3), transcript variant X3, mRNA	
41	XM_026288569.1	L	**S9866_174755**	Carassius auratus histone-lysine N-methyltransferase 2B-like (LOC113119259), mRNA	Q9UMN6
42	XM_026200903.1	L	**S9853_2824879**	Carassius auratus Krueppel-like factor 6 (LOC113042235), mRNA	Q99612
43	XM_026204651.1	L	**S9853_2824879**	Carassius auratus monocyte to macrophage differentiation factor 2-like (LOC113044565), transcript variant X3, mRNA	Q8IY49
	XR_003275882.1			Carassius auratus monocyte to macrophage differentiation factor 2-like (LOC113044565), transcript variant X4, misc_RNA	L0R6K8
44	XM_026254993.1	L	**S9951_65613**	Carassius auratus tRNA pseudouridine(38/39) synthase-like (LOC113084860), transcript variant X3, mRNA	Q9BZE2
					E9PNY6
					E9PRI9
45	XM_026229009.1	L	**S9966_92994**	Carassius auratus zyxin-like (LOC113060145), transcript variant X1, mRNA	Q15942
	XM_026229010.1			Carassius auratus zyxin-like (LOC113060145), transcript variant X2, mRNA	
46	EU693901.1	L	**S124_317291**	Channa argus rhamnose-binding lectin (RBL) gene, promoter region and complete cds	B3KWG0
47	XM_017498060.1	H	**S217_136844**	Cebus capucinus imitator aspartate beta-hydroxylase domain containing 2 (ASPHD2), transcript variant X1, mRNA	A0A024R1D0
	XM_017498061.1			Cebus capucinus imitator aspartate beta-hydroxylase domain containing 2 (ASPHD2), transcript variant X2, mRNA	Q6ICH7
48	XM_023373558.1	L	**S885_89904**	Centruroides sculpturatus ras-related protein M-Ras-like (LOC111629674), mRNA	O14807
49	XM_010121678.1	L	**S9966_92994**	Chlamydotis macqueenii zinc finger protein 800 (ZNF800), mRNA	Q2TB10
					A0A087WXB4
					Q49A13
					C9JHX9
					C9K0M9
					C9JLY3
					Q4G0T8
50	XM_014406203.2	L	**S885_89904**	Cimex lectularius FYVE, RhoGEF and PH domain-containing protein 4-like (LOC106673856), mRNA	Q96M96
51	XM_015858645.1	B	**S8943_473**	Coturnix japonica BEN domain containing 3 (BEND3), transcript variant X1, mRNA	Q5T5X7

	XM_015858647.1			Coturnix japonica BEN domain containing 3 (BEND3), transcript variant X2, mRNA	
	XM_015858648.1			Coturnix japonica BEN domain containing 3 (BEND3), transcript variant X3, mRNA	
	XM_015858649.1			Coturnix japonica BEN domain containing 3 (BEND3), transcript variant X4, mRNA	
52	AY551097.1	L	**S9853_2824879**	Crassostrea gigas fatty acid binding protein mRNA, complete cds	P05413
					Q01469
					P12104
					O15540
					P07148
					P15090
					P51161
53	XM_023918766.1	L	**S897_124935**	Cyanistes caeruleus GATA binding protein 3 (GATA3), transcript variant X1, mRNA	P23771
	XM_023918767.1			Cyanistes caeruleus GATA binding protein 3 (GATA3), transcript variant X2, mRNA	A0A2R8Y4T2
	XM_023918768.1			Cyanistes caeruleus GATA binding protein 3 (GATA3), transcript variant X3, mRNA	A0A2R8Y2A9
54	XM_015375577.1	L	**S10010_1012466**	Cyprinodon variegatus acetoacetyl-CoA synthetase (aacs), mRNA	Q86V21
					A0A024RBV2
					E7EW25
					Q49AJ4
					L8ECK1
55	XM_015394546.1	L	**S174_3611515**	Cyprinodon variegatus AT-rich interactive domain-containing protein 3B-like (LOC107097412), transcript variant X1, mRNA	H3BQ92
	XM_015394547.1			Cyprinodon variegatus AT-rich interactive domain-containing protein 3B-like (LOC107097412), transcript variant X2, mRNA	Q99856
	XM_015394549.1			Cyprinodon variegatus AT-rich interactive domain-containing protein 3B-like (LOC107097412), transcript variant X3, mRNA	Q8IVW6
	XM_015394550.1			Cyprinodon variegatus AT-rich interactive domain-containing protein 3B-like (LOC107097412), transcript variant X4, mRNA	
56	XM_015396192.1	B + H	**S368_129091**	Cyprinodon variegatus calcium binding protein 2 (cabp2), mRNA	Q9NPB3
					A0A1B0GW24
					F1T0K2
					F5H458
57	XM_015380986.1	L + B	**S9884_420423**	Cyprinodon variegatus centrosomal protein 63kDa (cep63), mRNA	Q96MT8
58	XM_015372153.1	L	**S844_58960**	Cyprinodon variegatus cytochrome P450 2K4-like (LOC107083153), transcript variant X1, mRNA	A0A146Y2K4

59	XM_015395499.1	L	**S9991_599077**	Cyprinodon variegatus gastrula zinc finger protein XlCGF57.1-like (LOC107098061), mRNA	Q9H9H3
60	XM_015383039.1	B + H	**S368_129091**	Cyprinodon variegatus guanine nucleotide binding protein (G protein), beta polypeptide 1-like (gnb1l), transcript variant X1, mRNA	Q9BYB4
	XM_015383040.1			Cyprinodon variegatus guanine nucleotide binding protein (G protein), beta polypeptide 1-like (gnb1l), transcript variant X2, mRNA	C9JPQ6
	XM_015383041.1			Cyprinodon variegatus guanine nucleotide binding protein (G protein), beta polypeptide 1-like (gnb1l), transcript variant X3, mRNA	
61	XM_015393779.1	L	**S7079_1123**	Cyprinodon variegatus junctional adhesion molecule B-like (LOC107096894), mRNA	P57087
	XM_015399137.1			Cyprinodon variegatus junctional adhesion molecule B-like (LOC107100593), mRNA	Q9BX67
62	XM_015376787.1	B	**S9889_1518953**	Cyprinodon variegatus membrane-associated phosphatidylinositol transfer protein 2-like (LOC107086052), mRNA	Q9BZ72
63	XM_015399020.1	H	**S1024_14784**	Cyprinodon variegatus microtubule associated protein 1S (map1s), mRNA	Q66K74
64	XM_015394625.1	L	**S703_83370**	Cyprinodon variegatus microtubule associated protein 6 (map6), transcript variant X3, mRNA	Q96JE9
65	XM_015374902.1	L	**S703_83370**	Cyprinodon variegatus nesprin-2-like (LOC107084854), mRNA	Q8WXH0
					A0A0C4DGK3
					A0A0A0MRE3
					G3V5X4
66	XM_015401906.1	L	**S703_83370**	Cyprinodon variegatus potassium channel subfamily K member 5-like (LOC107102543), mRNA	O95279
					A0A024RD32
					A0A0B6VPR3
67	XM_015368897.1	L	**S2023_14478**	Cyprinodon variegatus protein NLRC3-like (LOC107080691), mRNA	Q7RTR2
68	XM_015378696.1	L	**S10010_1012466**	Cyprinodon variegatus ribonucleoprotein, PTB-binding 2 (raver2), mRNA	Q9HCJ3
					E9PE10
69	XM_015370037.1	L + B	**S9899_1841030**	Cyprinodon variegatus syntaxin 2 (stx2), mRNA	P32856
70	XM_015400128.1	H	**S10016_468136**	Cyprinodon variegatus TBC1 domain family member 25 (tbc1d25), transcript variant X1, mRNA	Q3MII6
	XM_015400129.1			Cyprinodon variegatus TBC1 domain family member 25 (tbc1d25), transcript variant X2, mRNA	
71	XM_015376231.1	L	**S844_58960**	Cyprinodon variegatus wolframin-like (LOC107085724), transcript variant X1, mRNA	O76024
	XM_015376232.1			Cyprinodon variegatus wolframin-like (LOC107085724), transcript variant X2, mRNA	A0A0S2Z4V6
	XM_015376233.1			Cyprinodon variegatus wolframin-like (LOC107085724), transcript variant X3, mRNA	H0Y9G5
					A0A0E3H6T4

					B4DJ99
					X2CT56
72	XM_015374425.1	L	**S844_58956**	Cyprinodon variegatus X-ray repair complementing defective repair in Chinese hamster cells 4 (xrcc4), transcript variant X2, mRNA	Q13426
					A0A024RAP0
					A0A024RAL0
					A0A024RAQ8
73	XM_015391306.1	B	**S1073_18989**	Cyprinodon variegatus zinc finger protein 883-like (LOC107095288), mRNA	Q96MB7
74	XM_003199319.5	L	**S897_124935**	Danio rerio long-chain fatty acid transport protein 6-like (LOC568883), transcript variant X1, mRNA	Q9Y2P4
75	XR_002459143.1	L	**S10156_378391**	Danio rerio meiosis-specific nuclear structural 1 (mns1), transcript variant X1, misc_RNA	Q8NEH6
	XR_002459144.1			Danio rerio meiosis-specific nuclear structural 1 (mns1), transcript variant X2, misc_RNA	B3KQ70
76	BC168467.1	B	**S3317_962**	Danio rerio actin, alpha 1, skeletal muscle, mRNA (cDNA clone MGC:172132 IMAGE:8000653), complete cds	P68133
	BC065435.1			Danio rerio actin, alpha 1, skeletal muscle, mRNA (cDNA clone MGC:77653 IMAGE:6997034), complete cds	
77	NM_001080579.1	L	**S897_124935**	Danio rerio ArfGAP with coiled-coil, ankyrin repeat and PH domains 1 (acap1), mRNA	Q15027
					I3L0K9
					I3L268
78	BC077086.1	L	**S9929_1038014**	Danio rerio ATG5 autophagy related 5 homolog (S. cerevisiae), mRNA (cDNA clone MGC:100934 IMAGE:7145909), complete cds	
79	NM_194398.2	L	**S178_234532**	Danio rerio BCL2 like 10 (bcl2l10), mRNA	Q9HD36
					H0YMD5
80	NM_001040328.1	L	**S885_89904**	Danio rerio LCK proto-oncogene, Src family tyrosine kinase (lck), transcript variant 2, mRNA	P06239
81	BC064299.1	L	**S9929_1038014**	Danio rerio proliferating cell nuclear antigen, mRNA (cDNA clone MGC:77707 IMAGE:7000501), complete cds	P12004
82	NM_200324.1	L	**S9853_2824879**	Danio rerio unconventional SNARE in the ER 1 homolog (S. cerevisiae) (use1), mRNA	Q9NZ43
83	XM_004472646.2	L	**S9929_1038014**	Dasypus novemcinctus transmembrane protein 150C (TMEM150C), transcript variant X1, mRNA	B9EJG8
	XM_012530686.2			Dasypus novemcinctus transmembrane protein 150C (TMEM150C), transcript variant X2, mRNA	D6RAQ9
	XM_012530685.2			Dasypus novemcinctus transmembrane protein 150C (TMEM150C), transcript variant X3, mRNA	D6RDW6
	XM_023582847.1			Dasypus novemcinctus transmembrane protein 150C (TMEM150C), transcript variant X4, mRNA	
	XM_012530687.2			Dasypus novemcinctus transmembrane protein 150C (TMEM150C), transcript variant X5, mRNA	
84	XM_014618888.1	L	**S897_124935**	Dinoponera quadriceps splicing factor 3B subunit 3 (LOC106744262), transcript variant X1, mRNA	Q15393

	XM_014618897.1			Dinoponera quadriceps splicing factor 3B subunit 3 (LOC106744262), transcript variant X2, mRNA	
85	XM_026115261.1	L	**S9853_2824879**	Dromaius novaehollandiae probable ubiquitin carboxyl-terminal hydrolase FAF-X (LOC112992287), transcript variant X1, mRNA	Q93008
	XM_026115262.1			Dromaius novaehollandiae probable ubiquitin carboxyl-terminal hydrolase FAF-X (LOC112992287), transcript variant X2, mRNA	
86	XM_026104388.1	L	**S9966_776829**	Dromaius novaehollandiae tubby bipartite transcription factor (TUB), transcript variant X1, mRNA	P50607
	XM_026104389.1			Dromaius novaehollandiae tubby bipartite transcription factor (TUB), transcript variant X2, mRNA	
	XM_026104390.1			Dromaius novaehollandiae tubby bipartite transcription factor (TUB), transcript variant X3, mRNA	
	XM_026104391.1			Dromaius novaehollandiae tubby bipartite transcription factor (TUB), transcript variant X4, mRNA	
	XM_026104392.1			Dromaius novaehollandiae tubby bipartite transcription factor (TUB), transcript variant X5, mRNA	
	XM_026104393.1			Dromaius novaehollandiae tubby bipartite transcription factor (TUB), transcript variant X6, mRNA	
87	XM_027007299.1	L	**S9866_174755**	Electrophorus electricus zinc finger protein 423-like (LOC113575680), transcript variant X1, mRNA	Q2M1K9
	XM_027007300.1			Electrophorus electricus zinc finger protein 423-like (LOC113575680), transcript variant X2, mRNA	
	XM_027007301.1			Electrophorus electricus zinc finger protein 423-like (LOC113575680), transcript variant X3, mRNA	
	XM_027007302.1			Electrophorus electricus zinc finger protein 423-like (LOC113575680), transcript variant X4, mRNA	
	XM_027007303.1			Electrophorus electricus zinc finger protein 423-like (LOC113575680), transcript variant X5, mRNA	
	XM_027007304.1			Electrophorus electricus zinc finger protein 423-like (LOC113575680), transcript variant X6, mRNA	
88	GU984383.1	L	**S9929_1038014**	Epinephelus bruneus kisspeptin (KiSS2) mRNA, complete cds	Q15726
					A0A0D9SES6
89	AF124051.1	L	**S124_317291**	Fugu rubripes double stranded RNA adenosine deaminase RED2 gene, partial cds	Q9NS39
90	AF079311.1	B	**S9885_35456**	Fundulus heteroclitus aryl hydrocarbon receptor nuclear translocator 2 (ARNT2) mRNA, complete cds	Q9HBZ2
	NM_001309983.1			Fundulus heteroclitus aryl hydrocarbon receptor nuclear translocator 2 (arnt2), mRNA	X5DQN9
					A0A087WVE9
					Q86TN1
					X5DP31
					X5DP40
					X5D7T2
					X5D9H9

					X5DRE4<br>H0YKW1<br>X5D8U0<br>X5DP87
91	KM236111.1	B	**S10045_80975**	Fundulus heteroclitus estrogen receptor alpha (esr1) gene, complete cds	G4XH65<br>P03372<br>E5G0W7
92	XM_021323058.1	B + H	**S368_129091**	Fundulus heteroclitus ankyrin repeat domain 52 (ankrd52), mRNA	Q8NB46<br>L8EAS5
93	XM_012859214.2	L	**S132_473992**	Fundulus heteroclitus B-cell receptor CD22-like (LOC105923314), mRNA	P20273
94	XM_021315295.1	H	**S9951_762061**	Fundulus heteroclitus biotinidase-like (LOC105924216), mRNA	P43251
95	XM_021323537.1<br>XM_021323538.1<br>XM_021323539.1	L	**S470_275634**	Fundulus heteroclitus bone morphogenetic protein receptor type-2-like (LOC105935861), transcript variant X1, mRNA<br>Fundulus heteroclitus bone morphogenetic protein receptor type-2-like (LOC105935861), transcript variant X2, mRNA<br>Fundulus heteroclitus bone morphogenetic protein receptor type-2-like (LOC105935861), transcript variant X3, mRNA	Q13873
96	XM_021318945.1	L	**S470_275634**	Fundulus heteroclitus C-Jun-amino-terminal kinase-interacting protein 1-like (LOC105929632), partial mRNA	Q9UQF2
97	XM_021322757.1	H	**S10108_580435**	Fundulus heteroclitus calcium dependent secretion activator 2 (cadps2), mRNA	Q86UW7<br>C9IYE1<br>A0A087X1P3<br>F8W8P5<br>H0Y8B5
98	XM_021312860.1	L + B	**S1691_12502**	Fundulus heteroclitus carcinoembryonic antigen-related cell adhesion molecule 1-like (LOC110367333), mRNA	P13688
99	XM_021309557.1	B	**S9856_2465434**	Fundulus heteroclitus cationic amino acid transporter 3 (LOC105916243), mRNA	Q8WY07
100	XM_021308469.1<br>XM_021308470.1	L	**S470_275634**	Fundulus heteroclitus cerebellar degeneration-related protein 2-like (LOC105939930), transcript variant X1, mRNA<br>Fundulus heteroclitus cerebellar degeneration-related protein 2-like (LOC105939930), transcript variant X2, mRNA	P52569
101	XM_012859882.2	L	**S470_275634**	Fundulus heteroclitus cholinesterase 1-like (LOC105923969), mRNA	P06276
102	XM_012855450.2	B	**S4919_3133**	Fundulus heteroclitus cold shock domain-containing protein C2-like (LOC105919973), transcript variant X2, mRNA	Q9Y534<br>H7C4E7

103	XM_021313000.1	L	**S10103_490931**	Fundulus heteroclitus contactin-associated protein-like 2 (LOC105920987), mRNA	Q9UHC6
					Q02246
104	XM_012862948.2	H	**S105_467399**	Fundulus heteroclitus cytochrome P450 2P2 (LOC105926571), mRNA	Q9PTR2
105	XM_012859139.2	L	**S703_83370**	Fundulus heteroclitus DEAD-box helicase 51 (ddx51), mRNA	Q8N8A6
					F1T0L5
106	XM_012851665.2	L	**S216_297421**	Fundulus heteroclitus dehydrogenase/reductase SDR family member 11-like (LOC105916998), mRNA	Q6UWP2
107	XM_021321046.1	L	**S470_275634**	Fundulus heteroclitus discoidin domain receptor tyrosine kinase 2 (ddr2), mRNA	Q16832
					A0A024R906
					Q5T244
					Q5T245
					Q5T241
					H0Y570
108	XM_012875545.2	L	**S470_275634**	Fundulus heteroclitus dishevelled segment polarity protein 1 (dvl1), mRNA	O14640
					P54792
					Q5IS48
109	XM_012868134.2	B	**S4919_3133**	Fundulus heteroclitus endothelial cell adhesion molecule (esam), mRNA	Q96AP7
					F8WDW9
					C9JIE7
110	XR_002428333.1	B	**S60_401521**	Fundulus heteroclitus erbb2 interacting protein (erbin), transcript variant X6, misc_RNA	Q96RT1
					B4DIP2
					H0YA04
					H0Y9E8
111	XM_021312271.1	L	**S803_137883**	Fundulus heteroclitus filamin A interacting protein 1 like (filip1l), transcript variant X7, mRNA	P21333
	XM_021312272.1			Fundulus heteroclitus filamin A interacting protein 1 like (filip1l), transcript variant X8, mRNA	
112	XM_012862948	H	**S105_467399**	PREDICTED: Fundulus heteroclitus cytochrome P450 2F2 (LOC105926571), mRNA	A0A0U1RPL9
113	XM_021322207.1	L	**S470_275634**	Fundulus heteroclitus FYVE, RhoGEF and PH domain-containing protein 6-like (LOC105934005), transcript variant X1, mRNA	Q6ZV73
	XM_012873812.2			Fundulus heteroclitus FYVE, RhoGEF and PH domain-containing protein 6-like (LOC105934005), transcript variant X2, mRNA	
114	XM_012876512.2	L	**S10116_1813332**	Fundulus heteroclitus G protein subunit beta 1 like (gnb1l), transcript variant X1, mRNA	C9JPQ6

	XM_012876593.2			Fundulus heteroclitus G protein subunit beta 1 like (gnb1l), transcript variant X2, mRNA	Q9BYB4
115	XM_021310258.1	L	**S10116_1813332**	Fundulus heteroclitus G-patch domain containing 2 like (gpatch2l), mRNA	Q9NWQ4
116	XM_021320139.1	L	**S470_275634**	Fundulus heteroclitus gamma-glutamyl hydrolase (ggh), mRNA	Q92820
117	XM_021307273.1	L	**S897_124935**	Fundulus heteroclitus gap junction protein alpha 5 (gja5), transcript variant X1, mRNA	P36382
	XM_021307274.1			Fundulus heteroclitus gap junction protein alpha 5 (gja5), transcript variant X2, mRNA	A0A0B4J1Y3
118	XM_012873286.2	L	**S470_275634**	Fundulus heteroclitus glycerate kinase (glyctk), transcript variant X1, mRNA	Q8IVS8
	XM_021321928.1			Fundulus heteroclitus glycerate kinase (glyctk), transcript variant X2, mRNA	C9JA32
	XM_021321929.1			Fundulus heteroclitus glycerate kinase (glyctk), transcript variant X3, mRNA	C9J3N5
					A0A024R302
					A0A0C4DGA0
119	XM_012859274.2	L	**S9949_35040**	Fundulus heteroclitus glycine N-acyltransferase-like protein (LOC105923351), mRNA	Q6IB77
120	XM_012874883.2	L	**S10116_1813332**	Fundulus heteroclitus glycosyltransferase 8 domain containing 1 (glt8d1), mRNA	Q68CQ7
					C9J6X9
					C9J880
					A0A024R313
					C9JPK4
					C9JY96
					C9JNB0
					H7C4V6
121	XM_021314526.1	H	**S9860_2518005**	Fundulus heteroclitus Golgi apparatus protein 1-like (LOC105923080), transcript variant X1, mRNA	Q92896
122	XM_021316556.1	H	**S9867_2151582**	Fundulus heteroclitus golgin subfamily A member 6-like protein 22 (LOC110368556), transcript variant X1, mRNA	H0YM25
	XM_021316557.1			Fundulus heteroclitus golgin subfamily A member 6-like protein 22 (LOC110368556), transcript variant X2, mRNA	
	XR_002428179.1			Fundulus heteroclitus golgin subfamily A member 6-like protein 22 (LOC110368556), transcript variant X3, misc_RNA	
	XR_002428180.1			Fundulus heteroclitus golgin subfamily A member 6-like protein 22 (LOC110368556), transcript variant X4, misc_RNA	
123	XM_021307115.1	L	**S124_317291**	Fundulus heteroclitus GTPase IMAP family member 4-like (LOC105938037), mRNA	Q9NUV9
	XM_021310815.1			Fundulus heteroclitus GTPase IMAP family member 4-like (LOC110366389), mRNA	
124	XM_021313941.1	L	**S470_275634**	Fundulus heteroclitus helicase with zinc finger 2 (helz2), mRNA	Q9BYK8
125	XM_012873760.2	L	**S470_275634**	Fundulus heteroclitus high affinity choline transporter 1-like (LOC105933966), mRNA	Q9GZV3

126	XM_021320128.1	L	**S10116_1813332**	Fundulus heteroclitus IKBKB interacting protein (ikbip), transcript variant X1, mRNA	Q70UQ0
127	XM_012867057.2	L	**S9941_4782742**	Fundulus heteroclitus insulin receptor substrate 1-B-like (LOC105929332), transcript variant X2, mRNA	P06213
128	XM_021317315.1	L	**S10116_1813332**	Fundulus heteroclitus interleukin-17 receptor C (LOC105927307), mRNA	Q8NAC3
129	XM_012854238.2	L	**S10116_1813332**	Fundulus heteroclitus JNK1/MAPK8-associated membrane protein (jkamp), mRNA	Q9P055
					G3V2M4
					A0A087WVY6
					G3V4D0
					G3V2R2
					G3V372
130	XM_012865461.2	L	**S10116_1813332**	Fundulus heteroclitus kelch like family member 5 (klhl5), transcript variant X1, mRNA	Q96PQ7
	XM_021318032.1			Fundulus heteroclitus kelch like family member 5 (klhl5), transcript variant X2, mRNA	H0Y9Y5
					H0YAF6
					Q7Z6D5
					Q6PD75
					Q642I3
					A0A024R9V8
					L8E8A1
131	XM_012866162.2	H	**S10108_580435**	Fundulus heteroclitus kynurenine aminotransferase 1 (kyat1), mRNA	Q16773
					B7Z4W5
					Q5T276
					Q5T278
132	XM_021310058.1	L	**S470_275634**	Fundulus heteroclitus lamin-A-like (LOC105916913), transcript variant X1, mRNA	P02545
	XM_021310059.1			Fundulus heteroclitus lamin-A-like (LOC105916913), transcript variant X2, mRNA	
	XM_012851561.2			Fundulus heteroclitus lamin-A-like (LOC105916913), transcript variant X3, mRNA	
133	XM_012877853.2	L	**S470_275634**	Fundulus heteroclitus lipoprotein lipase-like (LOC105936851), mRNA	P06858
134	XM_012854276.2	L	**S470_275634**	Fundulus heteroclitus malignant fibrous histiocytoma amplified sequence 1 (mfhas1), transcript variant X1, mRNA	Q9Y4C4
					L8E7L4
135	XM_012853184.2	L + H	**S843_122311**	Fundulus heteroclitus mannose receptor C-type 1 (mrc1), transcript variant X1, mRNA	P22897

136	XM_012861657.2	L	**S10116_1813332**	Fundulus heteroclitus matrix metalloproteinase-15 (LOC105925688), mRNA	P51511
					Q7KZY0
137	XM_021316772.1	L	**S10116_1813332**	Fundulus heteroclitus matrix metalloproteinase-17-like (LOC105926619), mRNA	Q9ULZ9
138	XM_012856957.2	L	**S281_404055**	Fundulus heteroclitus methenyltetrahydrofolate synthetase (mthfs), transcript variant X1, mRNA	H3BMB9
					H3BN04
					P49914
					A0A0A6YYL1
					A0A0U1RQM3
					Q96EE9
139	XM_012870215.2	B	**S561_8990**	Fundulus heteroclitus MICAL-like protein 1 (LOC105931512), transcript variant X1, mRNA	Q8N3F8
	XM_021320280.1			Fundulus heteroclitus MICAL-like protein 1 (LOC105931512), transcript variant X2, mRNA	Q8IY33
	XM_021320281.1			Fundulus heteroclitus MICAL-like protein 1 (LOC105931512), transcript variant X3, mRNA	P51153
	XM_021320282.1			Fundulus heteroclitus MICAL-like protein 1 (LOC105931512), transcript variant X4, mRNA	B0QY91
	XM_012870216.2			Fundulus heteroclitus MICAL-like protein 1 (LOC105931512), transcript variant X5, mRNA	H0Y6J8
					B0QY86
140	XM_021316006.1	H	**S10010_923683**	Fundulus heteroclitus muscleblind-like protein 3 (LOC105925742), transcript variant X1, mRNA	Q9NUK0
	XM_021316007.1			Fundulus heteroclitus muscleblind-like protein 3 (LOC105925742), transcript variant X2, mRNA	B1AKI6
	XM_021316008.1			Fundulus heteroclitus muscleblind-like protein 3 (LOC105925742), transcript variant X3, mRNA	B1AKI4
	XM_012861721.2			Fundulus heteroclitus muscleblind-like protein 3 (LOC105925742), transcript variant X4, mRNA	B1AKI5
					B1AKI2
141	XM_012868298.2	L	**S470_275634**	Fundulus heteroclitus N-acetyllactosaminide beta-1,3-N-acetylglucosaminyltransferase 2-like (LOC105930205), mRNA	Q9Y2A9
					M0R199
					M0QX58
142	XM_012876676.2	H	**S10010_923683**	Fundulus heteroclitus neural-cadherin-like (LOC105936037), mRNA	P19022
143	XM_021323261.1	L	**S470_275634**	Fundulus heteroclitus neuroblast differentiation-associated protein AHNAK-like (LOC105935490), mRNA	E9PJZ0
					E9PLK4
					E9PKR9

					E9PJC6
					E9PQE3
					Q9BVU3
					Q96EC4
144	XM_012851721.2	B	**S385_310604**	Fundulus heteroclitus neuropeptide Y receptor type 1-like (LOC105917043), transcript variant X1, mRNA	B4DKL9
	XM_012851722.2			Fundulus heteroclitus neuropeptide Y receptor type 1-like (LOC105917043), transcript variant X2, mRNA	D6REY0
					D6R9D0
					D6RI97
					D6RHH6
					D6RC44
					P25929
145	XM_021322362.1	B	**S561_8990**	Fundulus heteroclitus nicotinamide/nicotinic acid mononucleotide adenylyltransferase 3 (LOC105934201), mRNA	D6RGH7
					D6RHV4
					D6R975
					D6REC8
					D6RGG8
					A0A2R8Y594
					A0A2R8YGL3
					A0A2R8YEU0
					A0A2R8YE08
					Q96T66
146	XM_021316272.1	L + H	**S9864_1470929**	Fundulus heteroclitus nuclear receptor subfamily 2 group C member 2 (nr2c2), mRNA	P49116
147	XM_012859216.2	L	**S4811_4274**	Fundulus heteroclitus oncoprotein induced transcript 3 (oit3), transcript variant X1, mRNA	Q8WWZ8
	XM_021314670.1			Fundulus heteroclitus oncoprotein induced transcript 3 (oit3), transcript variant X2, mRNA	
148	XM_012871695.2	B	**S561_8956**	Fundulus heteroclitus oocyte zinc finger protein XlCOF22-like (LOC105932495), mRNA	
149	XM_012875441.2	B	**S561_8990**	Fundulus heteroclitus origin recognition complex subunit 4-like (LOC105935160), mRNA	O43929
150	XM_012853337.2	B	**S10045_81009**	Fundulus heteroclitus p21 (RAC1) activated kinase 1 (pak1), mRNA	Q13153
151	XM_012868375.2	B	**S9885_35456**	Fundulus heteroclitus peroxisomal trans-2-enoyl-CoA reductase (pecr), mRNA	B4DJS2

					Q9BY49
152	XM_021319675.1	L	**S10116_1813332**	Fundulus heteroclitus phospholipid phosphatase 7 (inactive) (plpp7), transcript variant X2, mRNA	Q8NBV4
					X6R886
					A0A2R8Y4I9
153	XM_021319584.1	L	**S10150_201761**	Fundulus heteroclitus potassium channel tetramerization domain containing 15 (kctd15), transcript variant X1, mRNA	Q96SI1
	XM_012868679.2			Fundulus heteroclitus potassium channel tetramerization domain containing 15 (kctd15), transcript variant X2, mRNA	K7EN63
	XM_012868680.2			Fundulus heteroclitus potassium channel tetramerization domain containing 15 (kctd15), transcript variant X3, mRNA	V9GYY8
					K7EM48
					K7EQS3
					K7EPF0
					K7EIF1
154	XM_021323739.1	B	**S9917_1884759**	Fundulus heteroclitus potassium channel tetramerization domain containing 4 (kctd4), mRNA	Q8WVF5
155	XM_012859271.2	L	**S470_275634**	Fundulus heteroclitus potassium two pore domain channel subfamily K member 1 (kcnk1), mRNA	O00180
					A0A024R3T2
					Q5T5E6
156	XM_021310700.1	B	**S53_432183**	Fundulus heteroclitus protein phosphatase 1 regulatory subunit 15B-like (LOC105917785), mRNA	Q5SWA1
157	XM_012862438.2	L	**S470_275634**	Fundulus heteroclitus protein RIC-3-like (LOC105926210), mRNA	Q7Z5B4
					A0A0S2Z607
					E9PK46
158	XM_021313230.1	L	**S361_81605**	Fundulus heteroclitus quinolinate phosphoribosyltransferase (qprt), transcript variant X2, mRNA	Q15274
					H3BP73
159	XM_021308740.1	B	**S10045_81009**	Fundulus heteroclitus RAC-beta serine/threonine-protein kinase B (LOC105940337), transcript variant X1, mRNA	P31751
	XM_012882840.2			Fundulus heteroclitus RAC-beta serine/threonine-protein kinase B (LOC105940337), transcript variant X2, mRNA	C9JHS6
					J3QLS6
					M0QZK3
					J3QKW1
					J3QL45

					M0R283
					M0R275
					C9JIJ1
					A8MX96
					C9JC83
					E7EVP8
					J3KSY8
					J3KRI8
					C9JIF6
					C9J258
					A0A0A0MRF1
					M0QZW8
					J3KTC6
					J3KTP4
					A0A1B0GXA2
160	XM_012865928.2	L	**S10116_1813332**	Fundulus heteroclitus RNA-binding protein 38-like (LOC105928591), mRNA	Q9H0Z9
161	XM_021319315.1	L	**S10116_1813332**	Fundulus heteroclitus SAM domain-containing protein SAMSN-1-like (LOC105930089), transcript variant X1, mRNA	Q9NSI8
	XM_021319316.1			Fundulus heteroclitus SAM domain-containing protein SAMSN-1-like (LOC105930089), transcript variant X2, mRNA	S6FRS6
					A0A2R8Y4K8
162	XM_012880667.2	L	**S10075_823597**	Fundulus heteroclitus SAYSVFN motif domain containing 1 (saysd1), mRNA	Q9NPB0
163	XM_012880873.2	H	**S105_467399**	Fundulus heteroclitus scavenger receptor class A member 5 (scara5), transcript variant X1, mRNA	Q6ZMJ2
	XM_021307722.1			Fundulus heteroclitus scavenger receptor class A member 5 (scara5), transcript variant X2, mRNA	
164	XM_021309352.1	B	**S4128_2822**	Fundulus heteroclitus sorbin and SH3 domain-containing protein 1 (LOC105915919), transcript variant X1, mRNA	Q9BX66
	XM_021309354.1			Fundulus heteroclitus sorbin and SH3 domain-containing protein 1 (LOC105915919), transcript variant X2, mRNA	S4R303
	XM_021309355.1			Fundulus heteroclitus sorbin and SH3 domain-containing protein 1 (LOC105915919), transcript variant X3, mRNA	A0A0U1RQI5
	XM_021309356.1			Fundulus heteroclitus sorbin and SH3 domain-containing protein 1 (LOC105915919), transcript variant X4, mRNA	
	XM_021309357.1			Fundulus heteroclitus sorbin and SH3 domain-containing protein 1 (LOC105915919), transcript variant X5, mRNA	

	XM_021309358.1			Fundulus heteroclitus sorbin and SH3 domain-containing protein 1 (LOC105915919), transcript variant X6, mRNA	
	XM_021309359.1			Fundulus heteroclitus sorbin and SH3 domain-containing protein 1 (LOC105915919), transcript variant X7, mRNA	
	XM_021309360.1			Fundulus heteroclitus sorbin and SH3 domain-containing protein 1 (LOC105915919), transcript variant X8, mRNA	
165	XM_012875976.2	L	**S9929_413856**	Fundulus heteroclitus sphingosine-1-phosphate phosphatase 1-like (LOC105935524), mRNA	Q9BX95
166	XM_012869329.2	L	**S10116_1813332**	Fundulus heteroclitus SPRY domain-containing protein 3-like (LOC105930899), mRNA	Q6PJ21
167	XM_012879674.2	L	**S10116_1813332**	Fundulus heteroclitus sterol regulatory element binding transcription factor 2 (srebf2), mRNA	Q12772
					A0A024R1Q0
					A0A087X1T2
					G3V0I8
					H0Y7E5
168	XM_012859674.2	L	**S9869_217319**	Fundulus heteroclitus stonustoxin subunit alpha-like (LOC105923726), mRNA	A0A146SVJ8
					A0A146R4X3
					A0A146PFK2
					A0A146RM93
					A0A146R189
					A0A146PN29
					A0A146RQM2
					A0A146TTQ9
					A0A146PME0
					A0A146RKG4
					A0A146TYZ2
					A0A146PI85
					A0A146T2H2
					A0A146SKT8
					A0A146SAZ5
					A0A146RI68
					A0A146TS45
					A0A146PMN8

					A0A146Q2I0
					A0A146RJ50
					A0A146S8H6
					A0A146QVF2
					A0A146TAX8
					A0A146QCK6
					A0A146TV46
169	XM_012881138.1	L	**S1_2497139**	Fundulus heteroclitus sushi domain containing 1 (susd1), transcript variant X1, mRNA	Q6UWL2
	XM_012881219.1			Fundulus heteroclitus sushi domain containing 1 (susd1), transcript variant X2, mRNA	F8WAQ1
					H3BLV4
					H0Y6B2
					H0YCH6
170	XM_021318378.1	L + B	**S9899_1841030**	Fundulus heteroclitus syntaxin-2 (LOC105928768), transcript variant X1, mRNA	P32856
	XM_012866231.2			Fundulus heteroclitus syntaxin-2 (LOC105928768), transcript variant X2, mRNA	A0A348AY69
					J3KNU7
171	XM_021317112.1	L + H	**S42_249281**	Fundulus heteroclitus thyrotropin releasing hormone (trh), mRNA	A0A146Z4D1
					A0A147AU48
					A0A147B357
					A0A146ZXW2
					A0A146YGA5
					A0A147AVL5
					A0A146SP16
					A0A146VYC6
172	XM_012866902.2	B	**S4919_3133**	Fundulus heteroclitus transitional endoplasmic reticulum ATPase-like (LOC105929216), mRNA	P55072
					C9JUP7
					C9IZA5
173	XM_012861994.2	L	**S10010_1012466**	Fundulus heteroclitus transmembrane and coiled-coil domains 4 (tmco4), mRNA	Q5TGY1
					A0A075B6H3

					A0A024RAD2
					A0A024RA95
					A0A024RAA3
174	XM_012862605.2	B	**S9885_35456**	Fundulus heteroclitus uridine phosphorylase 1 (LOC105926333), mRNA	C9J486
					B4DND0
					C9K0J2
					F8WD51
					Q16831
175	XM_021307360.1	H	**S9915_1266854**	Fundulus heteroclitus vasoactive intestinal peptide receptor 1 (vipr1), mRNA	P32241
					C9JDT8
					A0A024R2N2
					C9JH33
					F2Z2U6
176	XM_012853036.2	B	**S9917_1884759**	Fundulus heteroclitus von Willebrand factor C domain containing 2 (vwc2), mRNA	B2RUY7
177	XM_012871962.2	L	**S2711_3408**	Fundulus heteroclitus wolframin ER transmembrane glycoprotein (wfs1), mRNA	Q2TAL6
178	XM_012855084.2	L	**S99_447176**	Fundulus heteroclitus WT1 interacting protein (LOC105919719), mRNA	A6NIX2
					Q96IZ0
179	XM_012871344.2	H	**S9915_1266854**	Fundulus heteroclitus zinc finger and BTB domain containing 14 (zbtb14), mRNA	O43829
180	XM_012867992.2	B	**S9885_35456**	Fundulus heteroclitus zinc finger DHHC-type containing 7 (zdhhc7), transcript variant X1, mRNA	Q9NXF8
	XM_021319233.1			Fundulus heteroclitus zinc finger DHHC-type containing 7 (zdhhc7), transcript variant X2, mRNA	H3BMI0
					H3BNQ9
181	XM_012851106.2	L	**S185_90462**	Fundulus heteroclitus zinc finger HIT-type containing 3 (znhit3), transcript variant X1, mRNA	Q15649
	XM_012851107.2			Fundulus heteroclitus zinc finger HIT-type containing 3 (znhit3), transcript variant X2, mRNA	A0A087X045
					A0A087WY54
					A0A024R0X8
					A0A087WTR0
					A0A087WY42
					A0A087X1G0

					A0A087WYI8
182	XM_012869702.2	L	**S10116_1813332**	Fundulus heteroclitus zinc finger protein 135-like (LOC105931150), transcript variant X1, mRNA	P52742
	XM_012869703.2			Fundulus heteroclitus zinc finger protein 135-like (LOC105931150), transcript variant X2, mRNA	
	XM_021320099.1			Fundulus heteroclitus zinc finger protein 135-like (LOC105931150), transcript variant X3, mRNA	
	XM_021313754.1			Fundulus heteroclitus zinc finger protein 260-like (LOC105921995), transcript variant X4, mRNA	
	XM_021313755.1			Fundulus heteroclitus zinc finger protein 260-like (LOC105921995), transcript variant X5, mRNA	
183	XM_021314095.1	L	**S470_275634**	Fundulus heteroclitus zinc finger protein OZF-like (LOC110367702), mRNA	Q15072
					Q9UJW8
184	AY040818.1	L	**S897_124935**	Galleria mellonella mucin-like protein mRNA, complete sequence	A0A146ZI98
					A0A146UW64
					A0A146UVM3
					A0A146VIR1
					A0A146NXA0
					A0A146YPL6
					A0A146NXW9
					A0A146VJH4
					A0A146VIT9
					A0A146VIX4
					A0A146VIF9
					A0A146VJX0
					A0A146VII5
					A0A146VIJ2
					A0A146VIH0
					A0A146VIF8
					A0A146ZK42
					A0A146ZHP4
					A0A146UXF5
					A0A146UWT9

					A0A146UW87
					A0A146YMT7
					A0A146N7F9
					A0A146YNW8
					A0A146YMT4
185	NM_204726.1	L	**S178_234532**	Gallus gallus caspase 6 (CASP6), mRNA	P55212
					D6RBM3
					D6RHU3
186	XM_016861023.1	L	**S9866_174755**	Gossypium hirsutum probable protein phosphatase 2C 76 (LOC107929561), transcript variant X1, mRNA	A0A1U8LPM0
	XM_016861024.1			Gossypium hirsutum probable protein phosphatase 2C 76 (LOC107929561), transcript variant X2, mRNA	A0A1U8JN63
					A0A1U8NE91
					A0A1U8JXL2
					A0A1U8LSX4
					A0A1U8JN81
187	XM_014421359.2	L	**S1100_29850**	Halyomorpha halys putative epidermal cell surface receptor (LOC106681176), transcript variant X1, mRNA	Q99527
	XM_014421360.2			Halyomorpha halys putative epidermal cell surface receptor (LOC106681176), transcript variant X2, mRNA	P13688
	XM_014421361.2			Halyomorpha halys putative epidermal cell surface receptor (LOC106681176), transcript variant X3, mRNA	P51864
188	XM_005945254.2	B	**S771_52821**	Haplochromis burtoni neuralized E3 ubiquitin protein ligase 4 (neurl4), transcript variant X1, mRNA	Q96JN8
	XM_014341031.1			Haplochromis burtoni neuralized E3 ubiquitin protein ligase 4 (neurl4), transcript variant X2, mRNA	I3L2W2
					I3L2Z9
					K7EPS7
					I3L100
189	XM_004865598.2	B	**S3317_962**	Heterocephalus glaber ELAV like RNA binding protein 3 (Elavl3), transcript variant X1, mRNA	Q14576
	XM_021244821.1			Heterocephalus glaber ELAV like RNA binding protein 3 (Elavl3), transcript variant X2, mRNA	Q96J71
	XM_004865599.2			Heterocephalus glaber ELAV like RNA binding protein 3 (Elavl3), transcript variant X3, mRNA	K7EPB5
					Q9H024
					L8E8Z0

190	XM_019882651.1	L	**S9966_776829**	Hippocampus comes B-cell lymphoma/leukemia 11A-like (LOC109523475), mRNA	Q9H165
					Q9C0K0
					A0A2U3TZJ5
					A0A0J9YXG2
					A0A2R8Y7B0
					A0A2R8Y2E8
					A0A2R8YDW6
					B4DT16
					B3KNX8
					B4DMK8
					B3KRW8
					A0A2R8YEK1
					A0A0J9YYJ9
					A0A0J9YY13
					A0A2R8YHH4
					A0A2R8YGT9
					A0A2R8Y7W4
					A0A2R8YDS7
					A0A2R8YCR5
191	XM_019636406.1	L	**S9966_92994**	Hipposideros armiger WW domain binding protein 1-like (WBP1L), transcript variant X1, mRNA	Q9NX94
	XM_019636407.1			Hipposideros armiger WW domain binding protein 1-like (WBP1L), transcript variant X2, mRNA	
	XM_019636408.1			Hipposideros armiger WW domain binding protein 1-like (WBP1L), transcript variant X3, mRNA	
192	NM_001350175.1	B	**S3317_962**	Homo sapiens ataxin 7 like 2 (ATXN7L2), transcript variant 1, mRNA	Q5T6C5
	NM_001350174.1			Homo sapiens ataxin 7 like 2 (ATXN7L2), transcript variant 3, mRNA	S4R3G5
					I6L9B5
193	BC049193.1	B	**S771_52821**	Homo sapiens DENN/MADD domain containing 2A, mRNA (cDNA clone IMAGE:4445131), partial cds	A2RUF6
194	DQ359746.1	L	**S1100_29850**	Homo sapiens ets family transcription factor ELF2C mRNA, complete cds, alternatively spliced	B0KYV4
195	NG_051966.1	L	**S9866_174755**	Homo sapiens fibroblast growth factor 12 (FGF12), RefSeqGene on chromosome 3	P61328

					C9JEN8
					C9JUK8
					C9JIN3
					Q99517
196	NG_007469.3	B	**S8943_473**	Homo sapiens glucosaminyl (N-acetyl) transferase 2 (I blood group) (GCNT2), RefSeqGene on chromosome 6	Q8N0V5
					A0A1W2PRW1
					A8K580
					B7ZBL3
197	BC053865.1	B	**S3317_962**	Homo sapiens KIAA1345 protein, mRNA (cDNA clone IMAGE:5204856)	Q9P2K1
198	BC146671.1	B	**S3317_962**	Homo sapiens mannosidase, endo-alpha, mRNA (cDNA clone MGC:164944 IMAGE:40148201), complete cds	Q5SRI9
					Q5VSG8
					X6R7A2
					E9PMU5
					H0YCZ3
					B4DW72
199	NM_054024.3	L	**S9966_92994**	Homo sapiens MIA SH3 domain ER export factor 2 (MIA2), transcript variant 9, mRNA	G3V599
					G3V5K6
					G3V3C4
					G3V4M1
					Q6PIE7
					Q96PC5
200	BC047761.1	B	**S3317_962**	Homo sapiens MKL/myocardin-like 2, mRNA (cDNA clone IMAGE:5741919), complete cds	Q9ULH7
					Q969V6
					B3KXK5
201	BC084547.1	H	**S9889_2454843**	Homo sapiens NCK-associated protein 1-like, mRNA (cDNA clone IMAGE:5451042), partial cds	P55160
					Q9Y2A7
					Q9HCH0
					O14513

					O14512
					P61586
					H7C1V4
					A0A0A0MS79
					H7C187
					A0A0A0MSE4
					A0A1W2PNT1
					F8W050
					C9JYL7
					A0A1W2PS86
					Q9BV52
					Q5XG97
					Q6P1M2
					B3KMK7
					B3KM75
					B2RA26
202	NG_028180.1	B	**S1223_43679**	Homo sapiens serpin family F member 1 (SERPINF1), RefSeqGene on chromosome 17	I3L3Z3
					I3L1U4
					I3L2R7
					I3L4Z0
					I3L425
					I3L4N7
					I3L107
					I3L4F9
					A0A0J9YXW2
					A0A0J9YXF9
					P36955
203	BC036298.1	L	**S9929_1038014**	Homo sapiens serpin peptidase inhibitor, clade H (heat shock protein 47), member 1, (collagen binding protein 1), mRNA (cDNA clone MGC:25195 IMAGE:4748644), complete cds	A0A024R5K8

					A8K259
204	NM_001042437.1	L	**S9929_1038014**	Homo sapiens ST3 beta-galactoside alpha-2,3-sialyltransferase 5 (ST3GAL5), transcript variant 2, mRNA	Q9UNP4
					A0A0S2Z4Q7
					A0A1W2PR45
					A0A1X7SBT2
					A0A1W2PRC6
					A0A1W2PQR0
					A0A0S2Z4S6
					A0A1W2PRD9
					A0A1W2PQQ6
					A0A1W2PRY1
					A0A1W2PPT1
					C9JYS9
					A0A1W2PQH5
					A0A1W2PQT6
					A0A1W2PR43
					A0A1W2PRT0
					A0A1W2PP90
					A0A1W2PQ01
					A0A1W2PNZ0
					A0A1W2PPQ6
					A0A1W2PQM6
					A0A1W2PP52
					A0A1W2PRP8
					A0A1W2PNR4
					A0A1W2PNV2
205	BC050384.1	L	**S9929_1038014**	Homo sapiens suppressor of Ty 3 homolog (S. cerevisiae), mRNA (cDNA clone MGC:51927 IMAGE:5758833), complete cds	Q7KZ85
					D3DTZ5

					A0A024RD67
					B4E0Q4
206	BC142727.1	L	**S1100_29850**	Homo sapiens ubiquitin specific peptidase 19, mRNA (cDNA clone IMAGE:40148933), partial cds	O94966
207	NM_145172.4	L	**S9929_1038014**	Homo sapiens WD repeat domain 63 (WDR63), transcript variant 1, mRNA	Q8IWG1
	NM_001288563.1			Homo sapiens WD repeat domain 63 (WDR63), transcript variant 2, mRNA	H0YEC9
					E9PLK2
					E9PLW4
					L8E9L7
208	GQ184290.1	L	**S9929_1038014**	Hyriopsis cumingii metallothionein mRNA, complete cds	P02795
					P13640
					P25713
					P04731
					P04732
					P04733
					P80297
					P80294
					P07438
					Q8N339
					A0A024R6R7
					P98164
					Q93083
					Q9Y4I5
					Q14872
					P47944
					P0DM35
					A1L3X4
					H3BRY8
					H3BSP9

					H3BSS0
					H3BQX6
					U3KQD7
					H3BR34
					H3BTG5
209	XM_017487033.1	L	**S9866_174755**	Ictalurus punctatus histone acetyltransferase KAT7-like (LOC108275911), transcript variant X2, mRNA	O95251
					D6RFZ5
210	XM_025011043.1	L	**S140_384361**	Kryptolebias marmoratus alpha-N-acetylgalactosaminide alpha-2,6-sialyltransferase 1-like (LOC108249294), transcript variant X4, mRNA	Q9NSC7
					K7EMB6
					G3XAD9
					K7EJA8
					K7EJC9
211	XM_017426954.2	L	**S140_384361**	Kryptolebias marmoratus contactin-4-like (LOC108242227), transcript variant X1, mRNA	Q8IWV2
	XM_017426955.2			Kryptolebias marmoratus contactin-4-like (LOC108242227), transcript variant X2, mRNA	Q9C0A0
					E9PDN6
					A0A087WTA1
					F5H107
					A0A0A0MR20
212	XM_017429141.2	L	**S361_81605**	Kryptolebias marmoratus leucine-rich repeat-containing protein 32-like (LOC108243584), transcript variant X1, mRNA	Q14392
	XM_017429139.2			Kryptolebias marmoratus leucine-rich repeat-containing protein 32-like (LOC108243584), transcript variant X2, mRNA	
213	XM_017426977.2	B	**S9888_703558**	Kryptolebias marmoratus MLLT3, super elongation complex subunit (mllt3), transcript variant X1, mRNA	P42568
	XM_025008252.1			Kryptolebias marmoratus MLLT3, super elongation complex subunit (mllt3), transcript variant X2, mRNA	A0A0S2Z448
	XM_025008253.1			Kryptolebias marmoratus MLLT3, super elongation complex subunit (mllt3), transcript variant X3, mRNA	A0A0S2Z449
					B1APT5
214	XM_017409427.2	L + H	**S42_249281**	Kryptolebias marmoratus thyrotropin releasing hormone (trh), mRNA	A0A146Z4D1
					A0A147AU48
					A0A147B357
					A0A146ZXW2

					A0A146YGA5
					A0A147AVL5
					A0A146SP16
					A0A146VYC6
215	XM_020639546.1	H	**S9874_1914529**	Labrus bergylta protein ADP-ribosylarginine hydrolase-like (LOC109988148), mRNA	P54922
					Q9NX46
					Q8NDY3
					A8K6X2
					B4E341
					C9JZW7
					X6RL45
216	XM_020633577.1	L	**S9887_384977**	Labrus bergylta SWI/SNF related, matrix associated, actin dependent regulator of chromatin, subfamily d, member 3 (smarcd3), transcript variant X1, mRNA	A0A090N8Z9
	XM_020633578.1			Labrus bergylta SWI/SNF related, matrix associated, actin dependent regulator of chromatin, subfamily d, member 3 (smarcd3), transcript variant X2, mRNA	C9JYI7
	XM_020633579.1			Labrus bergylta SWI/SNF related, matrix associated, actin dependent regulator of chromatin, subfamily d, member 3 (smarcd3), transcript variant X3, mRNA	H7C4E9
	XM_020633580.1			Labrus bergylta SWI/SNF related, matrix associated, actin dependent regulator of chromatin, subfamily d, member 3 (smarcd3), transcript variant X4, mRNA	F8WBJ3
	XM_020633581.1			Labrus bergylta SWI/SNF related, matrix associated, actin dependent regulator of chromatin, subfamily d, member 3 (smarcd3), transcript variant X5, mRNA	Q6STE5
217	XM_020633015.1	L + B	**S9899_1841030**	Labrus bergylta syntaxin-2-like (LOC109983340), transcript variant X1, mRNA	P32856
	XM_020633016.1			Labrus bergylta syntaxin-2-like (LOC109983340), transcript variant X2, mRNA	A0A348AY69
					J3KNU7
218	XM_020646415.1	L	**S9869_217319**	Labrus bergylta tetraspanin-1-like (LOC109993450), mRNA	O60635
219	XM_010744022.2	L + B	**S10017_3443539**	Larimichthys crocea gamma-aminobutyric acid receptor subunit alpha-5 (LOC104929488), transcript variant X1, mRNA	G3V2Q9
					G3V2K2
					G3V408
					B4E1A2
					B4DFX3
					G3V2G8
					G3V296
					G3V2Y5

					P31644
220	NM_001303358.1	L	**S178_234532**	Larimichthys crocea peroxisome proliferator activated receptor alpha (ppara), mRNA	Q07869
221	XM_018695334.1	L	**S9866_174755**	Lates calcarifer disks large-associated protein 1-like (LOC108896281), mRNA	O14490
					A8MXQ8
					A0A0A0MTP4
					A8MYR7
					G3V543
					Q6IS01
222	XM_018669148.1	L + B	**S9899_1841030**	Lates calcarifer syntaxin-2-like (LOC108878442), transcript variant X1, mRNA	A0A348AY69
	XM_018669149.1			Lates calcarifer syntaxin-2-like (LOC108878442), transcript variant X2, mRNA	J3KNU7
223	XM_017804047.1	L	**S9966_92994**	Lepidothrix coronata adducin 3 (ADD3), transcript variant X1, mRNA	Q9UEY8
	XM_017804048.1			Lepidothrix coronata adducin 3 (ADD3), transcript variant X2, mRNA	Q5VU08
	XM_017804049.1			Lepidothrix coronata adducin 3 (ADD3), transcript variant X3, mRNA	
	XM_017804050.1			Lepidothrix coronata adducin 3 (ADD3), transcript variant X4, mRNA	
	XM_017804051.1			Lepidothrix coronata adducin 3 (ADD3), transcript variant X5, mRNA	
224	EU346906.1	L	**S9929_1038014**	Limnonectes kuhlii clone DTWAPC-3 antimicrobial peptide precursor, mRNA, complete cds	
225	XM_021532730.1	L	**S9966_92994**	Lonchura striata domestica actin related protein 2/3 complex subunit 3 (ARPC3), mRNA	O15145
226	NM_001283244.1	L	**S9853_2824879**	Macaca fascicularis contactin-associated protein-like 4-like (LOC102123544), mRNA	Q9C0A0
					E9PDN6
					A0A087WTA1
					F5H107
					A0A0A0MR20
227	NM_001283306.1	L	**S9929_1038014**	Macaca fascicularis transmembrane protein 35A (TMEM35), mRNA	Q53FP2
					Q8NCS4
					B4DN33
228	KY474042.1	L	**S178_234532**	Macrobrachium rosenbergii Cathepsin B mRNA, complete cds	P07858
					P04080
229	XM_011967421.1	L	**S178_234532**	Mandrillus leucophaeus KH-type splicing regulatory protein (KHSRP), transcript variant X1, mRNA	A0A087WTP3

	XM_011967423.1			Mandrillus leucophaeus KH-type splicing regulatory protein (KHSRP), transcript variant X2, mRNA	M0R3J3
					Q92945
230	XM_026295451.1	H	**S9874_1914529**	Mastacembelus armatus solute carrier family 10 member 2 (slc10a2), mRNA	Q12908
	XM_026322167.1			Mastacembelus armatus syntaxin-2-like (LOC113139154), transcript variant X1, mRNA	A0A348AY69
	XM_026322168.1			Mastacembelus armatus syntaxin-2-like (LOC113139154), transcript variant X2, mRNA	J3KNU7
					Q8N8E1
231	XM_004567917.2	B	**S3317_962**	Maylandia zebra cysteine rich hydrophobic domain 2 (chic2), transcript variant X1, mRNA	D6RDW7
	XM_004567918.2			Maylandia zebra cysteine rich hydrophobic domain 2 (chic2), transcript variant X2, mRNA	H0Y8H1
					Q9UKJ5
232	XM_004555468.4	L	**S9853_2824879**	Maylandia zebra family with sequence similarity 126 member B (fam126b), transcript variant X1, mRNA	Q8IXS8
	XM_004555469.5			Maylandia zebra family with sequence similarity 126 member B (fam126b), transcript variant X2, mRNA	C9JTA1
	XM_004555470.5			Maylandia zebra family with sequence similarity 126 member B (fam126b), transcript variant X3, mRNA	C9JNS4
	XM_004555472.4			Maylandia zebra family with sequence similarity 126 member B (fam126b), transcript variant X4, mRNA	C9J115
233	XM_004564881.2	B	**S561_8990**	Maylandia zebra potassium voltage-gated channel subfamily A member 1 (LOC101481697), mRNA	Q09470
234	XM_024800937.1	L	**S533_118016**	Maylandia zebra putative nuclease HARBI1 (LOC112432382), mRNA	E9PK24
	XM_024801416.1			Maylandia zebra putative nuclease HARBI1 (LOC112433095), mRNA	E9PQI1
	XM_024805100.1			Maylandia zebra putative nuclease HARBI1 (LOC112435979), mRNA	
	XM_024805867.1			Maylandia zebra putative nuclease HARBI1 (LOC112436357), mRNA	
235	XM_014413520.3	B	**S561_8990**	Maylandia zebra zinc finger BED domain-containing protein 1-like (LOC106676602), mRNA	O96006
					C9JXP4
236	XM_025345589.1	L	**S178_234532**	Melanaphis sacchari formin-like protein CG32138 (LOC112598922), transcript variant X1, mRNA	O95466
					Q8IVF7
					Q96PY5
					F8W1F5
					K7EK60
					K7EJE6
					K7ERL1
					A0A0A0MR62

					C9IZY8
					K7EMY8
					F8VYL1
237	EF694540.2	L	**S9853_2824879**	Morus nigra isolate MCaM-1 calmodulin 1 mRNA, complete cds	P0DP23
238	NM_028288.5	L	**S9929_1038014**	Mus musculus cullin 4B (Cul4b), transcript variant 1, mRNA	Q13620
	NM_001110142.1			Mus musculus cullin 4B (Cul4b), transcript variant 2, mRNA	A6NE76
					K4DI93
239	BC022634.2	L	**S1100_29850**	Mus musculus cytochrome P450, family 2, subfamily c, polypeptide 65, mRNA (cDNA clone IMAGE:4207800), partial cds	P11712
					P33261
					P10632
					P33260
					O15503
					O00264
					P16435
					S5RV20
					Q16750
					S5RV29
					S5R8G8
					S5RXA4
					S5R8H1
					S5RG22
					B7Z1F5
					Q9UQ59
					G3V188
					E9PIW6
					E9PLI9
					Q9UEH3
					A0A087X125

					Q2XN56
					A0A2R8YF67
					A0A1X9PW77
					A0A1X9PW33
240	NM_011156.3	B	**S8943_473**	Mus musculus prolyl endopeptidase (Prep), mRNA	P48147
					H0Y5Y0
241	BC085143.1	L	**S9929_1038014**	Mus musculus serine (or cysteine) peptidase inhibitor, clade H, member 1, mRNA (cDNA clone MGC:109669 IMAGE:6415383), complete cds	Q53YY1
242	BC082807.1	L	**S9853_2824879**	Mus musculus trafficking protein, kinesin binding 1, mRNA (cDNA clone IMAGE:6402633), partial cds	Q9UPV9
243	XM_013053922.1	L	**S178_234532**	Mustela putorius furo KIAA1109 ortholog (KIAA1109), transcript variant X1, mRNA	H7C0G8
	XM_013053923.1			Mustela putorius furo KIAA1109 ortholog (KIAA1109), transcript variant X2, mRNA	H0Y781
	XM_013053924.1			Mustela putorius furo KIAA1109 ortholog (KIAA1109), transcript variant X3, mRNA	H3BLT5
	XM_013053925.1			Mustela putorius furo KIAA1109 ortholog (KIAA1109), transcript variant X4, mRNA	H7C0Y8
	XM_013053926.1			Mustela putorius furo KIAA1109 ortholog (KIAA1109), transcript variant X5, mRNA	H7C121
	XM_013053927.1			Mustela putorius furo KIAA1109 ortholog (KIAA1109), transcript variant X6, mRNA	H7C3N8
	XM_004747974.2			Mustela putorius furo KIAA1109 ortholog (KIAA1109), transcript variant X7, mRNA	H7C2X5
					L8ECG0
					Q2LD37
244	XM_005884976.2	L	**S897_124935**	Myotis brandtii thymosin beta-4 (LOC102243622), transcript variant X1, mRNA	O14604
					P62328
245	XM_024734237.1	L	**S9966_776829**	Neophocaena asiaeorientalis asiaeorientalis protein tyrosine phosphatase, receptor type D (PTPRD), transcript variant X1, mRNA	P23468
	XM_024734238.1			Neophocaena asiaeorientalis asiaeorientalis protein tyrosine phosphatase, receptor type D (PTPRD), transcript variant X2, mRNA	Q3KPI9
	XM_024734239.1			Neophocaena asiaeorientalis asiaeorientalis protein tyrosine phosphatase, receptor type D (PTPRD), transcript variant X3, mRNA	F5GWR7
	XM_024734241.1			Neophocaena asiaeorientalis asiaeorientalis protein tyrosine phosphatase, receptor type D (PTPRD), transcript variant X4, mRNA	Q2HXI4
	XM_024734242.1			Neophocaena asiaeorientalis asiaeorientalis protein tyrosine phosphatase, receptor type D (PTPRD), transcript variant X5, mRNA	C9J8S8
	XM_024734243.1			Neophocaena asiaeorientalis asiaeorientalis protein tyrosine phosphatase, receptor type D (PTPRD), transcript variant X6, mRNA	C9J6E4
	XM_024734244.1			Neophocaena asiaeorientalis asiaeorientalis protein tyrosine phosphatase, receptor type D (PTPRD), transcript variant X7, mRNA	A0A1B0GU15
	XM_024734245.1			Neophocaena asiaeorientalis asiaeorientalis protein tyrosine phosphatase, receptor type D (PTPRD), transcript variant X8, mRNA	A0A1Y0ZJV0

					A0A1Y0ZSN9
246	XM_009624257.2	L	**S9866_174755**	Nicotiana tomentosiformis ubiquitin carboxyl-terminal hydrolase 2 (LOC104113935), mRNA	O75604
247	XM_015970797.1	L	**S4811_4274**	Nothobranchius furzeri FYN-binding protein-like (LOC107392791), mRNA	A0A024R032
					D6RFJ5
					D6RER7
					D6RAE8
					A0A2R8YEE1
					D6RC38
					Q5VWT5
					O15117
248	XM_015966627.1	L	**S430_235378**	Nothobranchius furzeri polypeptide N-acetylgalactosaminyltransferase 13 (galnt13), transcript variant X3, mRNA	Q8IUC8
					A0A1L4BJC0
					A0A1L4BJA6
					A0A1L4BJA9
					A0A1L4BJA8
					A0A1L4BJ91
					A0A1L4BJB1
					A0A1L4BJB0
					X5DRI3
					H7C2I5
					Q68VJ0
					H7C0T6
					H7BZU4
					H7BZG2
					C9JLI4
					L8ECM1
249	XM_015972117.1	L + B	**S9899_1841030**	Nothobranchius furzeri syntaxin-2-like (LOC107393623), transcript variant X1, mRNA	A0A348AY69
	XM_015972118.1			Nothobranchius furzeri syntaxin-2-like (LOC107393623), transcript variant X2, mRNA	J3KNU7

					Q8N8E1
250	XM_015956815.1	B	**S9856_2465434**	Nothobranchius furzeri tensin 3 (tns3), transcript variant X1, mRNA	Q68CZ2
	XM_015956817.1			Nothobranchius furzeri tensin 3 (tns3), transcript variant X2, mRNA	C9JHU5
	XM_015956818.1			Nothobranchius furzeri tensin 3 (tns3), transcript variant X3, mRNA	C9JUW5
	XM_015956819.1			Nothobranchius furzeri tensin 3 (tns3), transcript variant X4, mRNA	C9JTD0
	XM_015956820.1			Nothobranchius furzeri tensin 3 (tns3), transcript variant X5, mRNA	C9JWN9
					H7BZ64
251	XM_015969238.1	L	**S703_83370**	Nothobranchius furzeri transient receptor potential cation channel subfamily M member 4-like (LOC107391796), transcript variant X1, mRNA	Q8TD43
252	XM_010788027.1	L	**S99_447176**	Notothenia coriiceps CDK5 regulatory subunit associated protein 1-like 1 (cdkal1), partial mRNA	P06239
					Q5VV42
253	XM_004405919.2	L	**S9966_776829**	Odobenus rosmarus divergens protein tyrosine phosphatase, receptor type, D (PTPRD), transcript variant X1, mRNA	P23468
	XM_012564335.1			Odobenus rosmarus divergens protein tyrosine phosphatase, receptor type, D (PTPRD), transcript variant X2, mRNA	Q3KPI9
	XM_004405920.2			Odobenus rosmarus divergens protein tyrosine phosphatase, receptor type, D (PTPRD), transcript variant X3, mRNA	F5GWR7
	XM_004405921.2			Odobenus rosmarus divergens protein tyrosine phosphatase, receptor type, D (PTPRD), transcript variant X4, mRNA	Q2HXI4
	XM_004405922.2			Odobenus rosmarus divergens protein tyrosine phosphatase, receptor type, D (PTPRD), transcript variant X5, mRNA	C9J8S8
	XM_004405923.2			Odobenus rosmarus divergens protein tyrosine phosphatase, receptor type, D (PTPRD), transcript variant X6, mRNA	C9J6E4
					A0A1B0GU15
					A0A1Y0ZJV0
					A0A1Y0ZSN9
254	XM_021619932.1	L + B	**S9899_1841030**	Oncorhynchus mykiss syntaxin-2-like (LOC110535060), mRNA	A0A348AY69
					J3KNU7
					Q8N8E1
255	XR_003218842.1	L	**S9951_65613**	Oreochromis niloticus coxsackievirus and adenovirus receptor homolog (LOC102079545), transcript variant X1, misc_RNA	O75970
	XR_002061251.2			Oreochromis niloticus coxsackievirus and adenovirus receptor homolog (LOC102079545), transcript variant X2, misc_RNA	
256	XM_013273808.3	H	**S217_136844**	Oreochromis niloticus cystic fibrosis transmembrane conductance regulator (cftr), transcript variant X1, mRNA	P13569
	XM_003449170.5			Oreochromis niloticus cystic fibrosis transmembrane conductance regulator (cftr), transcript variant X2, mRNA	A0A024R730
					Q20BJ8

					Q20BI4
					Q20BH0
					Q2I0A9
					Q2I0A3
					Q20BI6
257	XM_025910860.1	L	**S2711_3408**	Oreochromis niloticus opioid-binding protein/cell adhesion molecule homolog (LOC100703630), transcript variant X1, mRNA	Q14982
	XM_019363978.2			Oreochromis niloticus opioid-binding protein/cell adhesion molecule homolog (LOC100703630), transcript variant X2, mRNA	Q5IS61
	XM_005454557.4			Oreochromis niloticus opioid-binding protein/cell adhesion molecule homolog (LOC100703630), transcript variant X3, mRNA	A8K0Y0
					B7ZLQ0
					Q6B0I4
					B2CZX3
258	XM_005458391.4	L	**S9888_466590**	Oreochromis niloticus protein FAM19A2 (LOC100704992), transcript variant X1, mRNA	F8VZY8
	XM_025907347.1			Oreochromis niloticus protein FAM19A2 (LOC100704992), transcript variant X2, mRNA	F8VVF9
					F8VWB2
					A0A0C4DGI5
					F8VVD7
					Q8N3H0
259	XM_019367491.2	H	**S1043_75350**	Oreochromis niloticus putative nuclease HARBI1 (LOC100692127), mRNA	E9PK24
	XM_019349300.1			Oreochromis niloticus putative nuclease HARBI1 (LOC109196088), mRNA	E9PQI1
	XM_019361975.2			Oreochromis niloticus putative nuclease HARBI1 (LOC109202968), mRNA	
260	XM_005450780.4	L	**S178_234532**	Oreochromis niloticus transcription factor SOX-4 (LOC100692790), mRNA	Q06945
261	AB270897.1	B	**S561_8990**	Oreochromis niloticus MHC class IA antigen UBA1, UBA2, UAA1 genes, partial cds, UAA3 and UAA2 pseudogenes, UAA4, UAA5 and UAA6 pseudogene fragments	Q95460
262	AB649032.1	B	**S561_8990**	Oreochromis niloticus vasa gene, clone: b04TI071H03	Q9NQI0
263	AB909496.1	B	**S371_298559**	Oryzias dancena Gpr112, Map7d2, Sox3, P2ry4, Inppl1b, Arr3 genes for G protein-coupled receptor 112, MAP7 domain containing 2, SRY-box containing transcription factor 3, G-protein coupled pyrimidinergic receptor P2Y4, inositol polyphosphate phosphatase-like 1b, arrestin 3, complete cds, chromosome X	Q8IZF6
					Q96T17
					A0A0M4F6E1
					A0A0M4FLI9

					A0A0K1JS24
					P41225
					P51582
					C6G7W3
					C6G7W4
					P36575
					D6RCT3
					A0A087WWQ5
264	XM_023957995.1	H	**S9889_2454843**	Oryzias latipes FAT atypical cadherin 1 (fat1), transcript variant X1, mRNA	H0Y9H4
	XM_023958002.1			Oryzias latipes FAT atypical cadherin 1 (fat1), transcript variant X2, mRNA	H0Y9C8
	XM_023958005.1			Oryzias latipes FAT atypical cadherin 1 (fat1), transcript variant X3, mRNA	H0Y8F5
	XM_023958007.1			Oryzias latipes FAT atypical cadherin 1 (fat1), transcript variant X4, mRNA	D6RHE6
	XM_023958011.1			Oryzias latipes FAT atypical cadherin 1 (fat1), transcript variant X5, mRNA	L8E7S1
	XM_023958018.1			Oryzias latipes FAT atypical cadherin 1 (fat1), transcript variant X6, mRNA	D6RCE4
	XM_023958025.1			Oryzias latipes FAT atypical cadherin 1 (fat1), transcript variant X7, mRNA	A0A087WVP1
					Q14517
265	XM_023954283.1	B	**S371_298559**	Oryzias latipes occludin (LOC101169436), mRNA	Q16625
					A0A0G2JMZ8
266	XM_004066447.4	B	**S1073_18989**	Oryzias latipes T-cell surface glycoprotein CD3 zeta chain (LOC101175223), transcript variant X1, mRNA	P20963
	XM_011482744.3			Oryzias latipes T-cell surface glycoprotein CD3 zeta chain (LOC101175223), transcript variant X2, mRNA	P14079
267	XM_011480878.3	B	**S1073_18989**	Oryzias latipes tax1-binding protein 1 homolog A (LOC101173317), transcript variant X1, mRNA	P03409
	XM_020707130.2			Oryzias latipes tax1-binding protein 1 homolog A (LOC101173317), transcript variant X2, mRNA	P0C213
					P0C222
268	XM_011484681.3	B	**S371_298559**	Oryzias latipes zinc transporter ZIP9 (LOC101163600), mRNA	Q9NUM3
269	XM_024275231.1	L + B	**S9899_1841030**	Oryzias melastigma syntaxin-2-like (LOC112148272), mRNA	A0A348AY69
					J3KNU7
					Q8N8E1
					O75970

270	DQ084040.1	L	**S1100_29850**	Oxyuranus scutellatus 60S ribosomal protein L13a mRNA, partial cds	P40429
271	MF403004.1	L	**S9966_92994**	Palaemon carinicauda beta carbonic anhydrase mRNA, complete cds	P23280
					P00918
					P00915
					P22748
					P07451
					O43570
					Q16790
					Q9ULX7
					P35219
					P43166
					Q8N1Q1
272	XM_024929407.1	B	**S8943_473**	Pan paniscus tripartite motif containing 33 (TRIM33), mRNA	A0A024R0F6
					H0Y612
					Q9UPN9
273	XM_024354201.1	H	**S217_136844**	Pan troglodytes ENAH, actin regulator (ENAH), transcript variant X1, mRNA	Q8N8S7
	XM_024354219.1			Pan troglodytes ENAH, actin regulator (ENAH), transcript variant X10, mRNA	A0A0U1RRM6
	XM_016939738.2			Pan troglodytes ENAH, actin regulator (ENAH), transcript variant X11, mRNA	A0A075B6E5
	XM_016939742.2			Pan troglodytes ENAH, actin regulator (ENAH), transcript variant X12, mRNA	A0A0U1RQP7
	XM_016939762.2			Pan troglodytes ENAH, actin regulator (ENAH), transcript variant X13, mRNA	A0A097PIC4
	XM_016939767.2			Pan troglodytes ENAH, actin regulator (ENAH), transcript variant X14, mRNA	
	XM_016939773.2			Pan troglodytes ENAH, actin regulator (ENAH), transcript variant X15, mRNA	
	XM_016939783.2			Pan troglodytes ENAH, actin regulator (ENAH), transcript variant X16, mRNA	
	XM_016939785.2			Pan troglodytes ENAH, actin regulator (ENAH), transcript variant X17, mRNA	
	XM_016939788.2			Pan troglodytes ENAH, actin regulator (ENAH), transcript variant X18, mRNA	
	XM_024354202.1			Pan troglodytes ENAH, actin regulator (ENAH), transcript variant X2, mRNA	
	XM_024354210.1			Pan troglodytes ENAH, actin regulator (ENAH), transcript variant X4, mRNA	
	XM_024354211.1			Pan troglodytes ENAH, actin regulator (ENAH), transcript variant X5, mRNA	
	XM_024354212.1			Pan troglodytes ENAH, actin regulator (ENAH), transcript variant X6, mRNA	

	XM_024354215.1			Pan troglodytes ENAH, actin regulator (ENAH), transcript variant X8, mRNA	
	XM_024354218.1			Pan troglodytes ENAH, actin regulator (ENAH), transcript variant X9, mRNA	
274	XM_026929900.1	L	**S9906_191627**	Pangasianodon hypophthalmus actin related protein 2/3 complex subunit 1A (arpc1a), mRNA	Q92747
					E9PF58
					Q75MY0
275	XM_026909765.1	L	**S10156_378391**	Pangasianodon hypophthalmus NLR family CARD domain-containing protein 3-like (LOC113523735), transcript variant X5, mRNA	Q7RTR2
					H3BLT9
					A0A087WZ24
276	XM_026913004.1	L	**S9941_4782742**	Pangasianodon hypophthalmus protein-lysine methyltransferase METTL21E-like (LOC113526176), transcript variant X1, mRNA	A6NDL7
	XM_026913005.1			Pangasianodon hypophthalmus protein-lysine methyltransferase METTL21E-like (LOC113526176), transcript variant X2, mRNA	
277	XM_019445039.1	L	**S885_89904**	Panthera pardus solute carrier family 16 member 12 (SLC16A12), mRNA	Q6ZSM3
278	XM_020093697.1	L + B	**S9899_1841030**	Paralichthys olivaceus syntaxin-2-like (LOC109633677), transcript variant X1, mRNA	A0A348AY69
	XM_020093698.1			Paralichthys olivaceus syntaxin-2-like (LOC109633677), transcript variant X2, mRNA	J3KNU7
	XM_020093699.1			Paralichthys olivaceus syntaxin-2-like (LOC109633677), transcript variant X3, mRNA	Q8N8E1
					O75970
					Q12955
279	HM100106.1	L	**S9853_2824879**	Paralichthys olivaceus transcription factor PU.1 mRNA, complete cds, alternatively spliced	P17947
					F5H3K6
					F5GZ94
					Q13318
280	XM_023816282.1	H	**S9889_2454843**	Paramormyrops kingsleyae lysine methyltransferase 2A (kmt2a), mRNA	Q03164
282	XM_023803392.1	L	**S468_2218143**	Paramormyrops kingsleyae SLAM family member 5-like (LOC111839455), mRNA	Q9UIB8
283	XM_006977726.2	L	**S9866_174755**	Peromyscus maniculatus bairdii zyg-11 family member B, cell cycle regulator (Zyg11b), mRNA	Q9C0D3
					A8DPD7
284	XM_023191319.2	B	**S3317_962**	Piliocolobus tephrosceles solute carrier family 30 member 7 (SLC30A7), transcript variant X1, mRNA	Q8NEW0
	XM_023191320.2			Piliocolobus tephrosceles solute carrier family 30 member 7 (SLC30A7), transcript variant X2, mRNA	H0Y362
285	XM_016679628.1	B + H	**S368_129091**	Poecilia formosa 2-aminoethanethiol dioxygenase-like (LOC103149020), transcript variant X1, mRNA	Q96SZ5

	XM_016679629.1			Poecilia formosa 2-aminoethanethiol dioxygenase-like (LOC103149020), transcript variant X2, mRNA	
286	XM_007541956.2	L	**S1062_14945**	Poecilia formosa butyrophilin subfamily 2 member A1-like (LOC103130540), transcript variant X1, mRNA	Q7KYR7
	XM_016666006.1			Poecilia formosa butyrophilin subfamily 2 member A1-like (LOC103130540), transcript variant X2, mRNA	H7BYC3
	XM_016666007.1			Poecilia formosa butyrophilin subfamily 2 member A1-like (LOC103130540), transcript variant X3, mRNA	H7C542
	XM_016679642.1			Poecilia formosa butyrophilin subfamily 2 member A1-like (LOC103149032), mRNA	C9JNC3
287	XM_007567174.2	L	**S9941_4782742**	Poecilia formosa carnitine O-acetyltransferase-like (LOC103148412), transcript variant X1, mRNA	P43155
288	XM_016671260.1	L	**S9941_4782742**	Poecilia formosa lysine-specific demethylase 4C-like (LOC103137779), transcript variant X5, mRNA	Q9H3R0
					F8WCN1
					C9J879
					A0A0A0MSR6
					B0QZ60
289	XM_007567329.2	L	**S703_83370**	Poecilia formosa mitochondrial uncoupling protein 2-like (LOC103148519), mRNA	P55851
					A0A024R5N5
					F5GX45
					H0YFR8
					H0YFQ0
					F5H312
290	XM_007558891.2	L	**S2711_3408**	Poecilia formosa mitogen-activated protein kinase 10 (mapk10), transcript variant X1, mRNA	P53779
	XM_016674757.1			Poecilia formosa mitogen-activated protein kinase 10 (mapk10), transcript variant X2, mRNA	D6RCB1
	XM_016674758.1			Poecilia formosa mitogen-activated protein kinase 10 (mapk10), transcript variant X3, mRNA	A8MWW6
	XM_016674759.1			Poecilia formosa mitogen-activated protein kinase 10 (mapk10), transcript variant X4, mRNA	D6RJF9
291	XM_007577262.2	L	**S2711_3408**	Poecilia formosa niban-like protein 1 (LOC103155545), transcript variant X1, mRNA	Q96TA1
	XM_016663646.1			Poecilia formosa niban-like protein 1 (LOC103155545), transcript variant X2, mRNA	
	XM_016663647.1			Poecilia formosa niban-like protein 1 (LOC103155545), transcript variant X3, mRNA	
292	XM_007561714.2	L	**S174_3611515**	Poecilia formosa ras-related protein Rab-38-like (LOC103144640), mRNA	P57729
					H0YDB7
					H0YEA4
293	XM_007540708.2	L	**S174_3611515**	Poecilia formosa round spermatid basic protein 1-like (LOC103129554), mRNA	Q5VWQ0

					Q6PCB5
					C9JM20
					H7C2D3
					A0A0C4DH79
294	XM_007572875.1	L	**S1_2497140**	Poecilia formosa sushi domain containing 1 (susd1), mRNA	F8WAQ1
					H3BLV4
					H0Y6B2
					H0YCH6
295	XM_007567117.2	L + B	**S9899_1841030**	Poecilia formosa syntaxin-2 (LOC103148388), transcript variant X1, mRNA	A0A348AY69
	XM_007567118.2			Poecilia formosa syntaxin-2 (LOC103148388), transcript variant X2, mRNA	J3KNU7
					Q8N8E1
					O75970
					Q12955
296	XM_007562325.2	L + H	**S42_249281**	Poecilia formosa thyrotropin releasing hormone (trh), transcript variant X1, mRNA	A0A146Z4D1
	XM_016676477.1			Poecilia formosa thyrotropin releasing hormone (trh), transcript variant X2, mRNA	A0A147AU48
					A0A147B357
					A0A146ZXW2
					A0A146YGA5
					A0A147AVL5
					A0A146SP16
					A0A146VYC6
297	XM_007551204.2	L	**S2711_3408**	Poecilia formosa transmembrane protein 26 (tmem26), mRNA	H7BXI3
					Q6ZUK4
298	XM_015052885.1	B + H	**S368_129091**	Poecilia latipinna 2-aminoethanethiol dioxygenase-like (LOC106959984), transcript variant X1, mRNA	Q96SZ5
	XM_015052886.1			Poecilia latipinna 2-aminoethanethiol dioxygenase-like (LOC106959984), transcript variant X2, mRNA	Q7KYR7
299	XM_015022562.1	L	**S1062_14945**	Poecilia latipinna butyrophilin subfamily 2 member A1-like (LOC106939965), mRNA	H7BYC3
	XM_015032189.1			Poecilia latipinna butyrophilin subfamily 2 member A1-like (LOC106947292), mRNA	H7C542
					C9JNC3
300	XM_015024325.1	L	**S1062_14945**	Poecilia latipinna carnitine O-acetyltransferase-like (LOC106941341), transcript variant X1, mRNA	P43155

301	XM_015041198.1	H	**S217_136844**	Poecilia latipinna integrin beta 6 (itgb6), transcript variant X1, mRNA	P18564
	XM_015041199.1			Poecilia latipinna integrin beta 6 (itgb6), transcript variant X2, mRNA	A0A087WXP3
	XM_015041200.1			Poecilia latipinna integrin beta 6 (itgb6), transcript variant X3, mRNA	E9PEE8
	XM_015041201.1			Poecilia latipinna integrin beta 6 (itgb6), transcript variant X4, mRNA	F8WBJ8
302	XM_015043850.1	H	**S217_136844**	Poecilia latipinna lysine-specific demethylase 4C-like (LOC106954381), transcript variant X6, mRNA	Q9H3R0
					F8WCN1
					C9J879
					A0A0A0MSR6
					B0QZ60
303	XM_015041133.1	L	**S703_83370**	Poecilia latipinna mitochondrial uncoupling protein 2-like (LOC106952793), mRNA	P55851
					A0A024R5N5
					F5GX45
					H0YFR8
					H0YFQ0
					F5H312
304	XM_015045411.1	L	**S703_83370**	Poecilia latipinna mucin-5AC-like (LOC106955308), mRNA	P98088
					T1R7M6
					T1R7A8
					T1R7N1
					T1R7N3
					Q96DU6
305	XM_015019529.1	L	**S2711_3408**	Poecilia latipinna niban-like protein 1 (LOC106937818), transcript variant X1, mRNA	Q96TA1
	XM_015019531.1			Poecilia latipinna niban-like protein 1 (LOC106937818), transcript variant X2, mRNA	
	XM_015019532.1			Poecilia latipinna niban-like protein 1 (LOC106937818), transcript variant X3, mRNA	
306	XM_015035724.1	L	**S9966_92994**	Poecilia latipinna phosphatidylinositol-4,5-bisphosphate 3-kinase, catalytic subunit delta (pik3cd), transcript variant X1, mRNA	O00329
	XM_015035725.1			Poecilia latipinna phosphatidylinositol-4,5-bisphosphate 3-kinase, catalytic subunit delta (pik3cd), transcript variant X2, mRNA	Q5SR50
					F8W9P4
					A0A2K8FKV1
					A7E2E0

					B7ZM44
					A0A2K8FKT1
					A0A2K8FKR1
					A0A2K8FKQ1
307	XM_015055566.1	L	**S174_3611515**	Poecilia latipinna round spermatid basic protein 1-like (LOC106961678), mRNA	Q5VWQ0
					Q6PCB5
					C9JM20
					H7C2D3
					A0A0C4DH79
308	XM_015015672.1	L + B	**S9899_1841030**	Poecilia latipinna syntaxin-2-like (LOC106935276), transcript variant X1, mRNA	P43155
	XM_015015673.1			Poecilia latipinna syntaxin-2-like (LOC106935276), transcript variant X2, mRNA	A0A147AU48
309	XM_015033403.1	L + H	**S42_249281**	Poecilia latipinna thyrotropin-releasing hormone (trh), transcript variant X1, mRNA	A0A147B357
	XM_015033404.1			Poecilia latipinna thyrotropin-releasing hormone (trh), transcript variant X2, mRNA	A0A146ZXW2
					A0A146YGA5
					A0A147AVL5
					A0A146SP16
					A0A146VYC6
310	XM_014975057.1	H	**S490_150742**	Poecilia mexicana 1-phosphatidylinositol 4,5-bisphosphate phosphodiesterase delta-4-like (LOC106908831), mRNA	Q9BRC7
					H7C237
					H7C281
					C9JAE4
					F2Z3H8
311	XM_014984307.1	B + H	**S368_129091**	Poecilia mexicana 2-aminoethanethiol dioxygenase-like (LOC106916187), transcript variant X1, mRNA	Q7KYR7
	XM_014984316.1			Poecilia mexicana 2-aminoethanethiol dioxygenase-like (LOC106916187), transcript variant X2, mRNA	
312	XM_014984789.1	L	**S9853_2824879**	Poecilia mexicana ALG3, alpha-1,3- mannosyltransferase (alg3), transcript variant X1, mRNA	Q92685
	XM_014984790.1			Poecilia mexicana ALG3, alpha-1,3- mannosyltransferase (alg3), transcript variant X2, mRNA	C9J7S5
					H7C0X4
					8WE30

					H7BZZ2
					F8WF93
313	XM_015001947.1	L	**S1062_14945**	Poecilia mexicana butyrophilin subfamily 1 member A1-like (LOC106926811), mRNA	H7BYC3
	XM_015001954.1			Poecilia mexicana butyrophilin subfamily 1 member A1-like (LOC106926817), mRNA	H7C542
314	XM_014976611.1	L	**S1062_14945**	Poecilia mexicana butyrophilin-like protein 2 (LOC106910057), transcript variant X1, mRNA	C9JNC3
	XM_014976612.1			Poecilia mexicana butyrophilin-like protein 2 (LOC106910057), transcript variant X2, mRNA	
	XM_014976613.1			Poecilia mexicana butyrophilin-like protein 2 (LOC106910057), transcript variant X3, mRNA	
315	XM_015002937.1	L	**S9941_4782742**	Poecilia mexicana carnitine O-acetyltransferase-like (LOC106927398), transcript variant X1, mRNA	P43155
316	XM_014981515.1	L	**S9869_217319**	Poecilia mexicana interleukin-18 receptor 1-like (LOC106914618), mRNA	O95256
					Q14116
					O95998
317	XM_014988676.1	L	**S9941_4782742**	Poecilia mexicana lysine-specific demethylase 4C-like (LOC106918705), transcript variant X5, mRNA	Q9H3R0
					F8WCN1
					C9J879
					A0A0A0MSR6
318	XM_015003443.1	L	**S703_83370**	Poecilia mexicana mitochondrial uncoupling protein 2-like (LOC106927699), mRNA	B0QZ60
					P55851
					A0A024R5N5
					F5GX45
					H0YFR8
					H0YFQ0
					F5H312
319	XM_014971399.1	L	**S2711_3408**	Poecilia mexicana niban-like protein 1 (LOC106906223), transcript variant X1, mRNA	Q96TA1
	XM_014971400.1			Poecilia mexicana niban-like protein 1 (LOC106906223), transcript variant X2, mRNA	
320	XM_014995514.1	L	**S10010_1012466**	Poecilia mexicana p21 protein (Cdc42/Rac)-activated kinase 4 (pak4), transcript variant X1, mRNA	A0A024R0J1
	XM_014995515.1			Poecilia mexicana p21 protein (Cdc42/Rac)-activated kinase 4 (pak4), transcript variant X2, mRNA	A0A024R0L8
					M0R1R1
					M0R0L9

					M0R2X4
					M0R3G6
					B4DUG0
321	XM_014982195.1	L	**S9866_174755**	Poecilia mexicana RAB11 family interacting protein 4 (class II) (rab11fip4), transcript variant X1, mRNA	Q86YS3
	XM_014982231.1			Poecilia mexicana RAB11 family interacting protein 4 (class II) (rab11fip4), transcript variant X5, mRNA	K7EL58
					J3QKR9
					K7EMK3
					J3QLM3
					L8ECL7
322	XM_015002153.1	L	**S174_3611515**	Poecilia mexicana ras-related protein Rab-38-like (LOC106926937), mRNA	P57729
					H0YDB7
					H0YEA4
323	XM_015010549.1	L + B	**S9899_1841030**	Poecilia mexicana syntaxin-2-like (LOC106932068), transcript variant X1, mRNA	P43155
	XM_015010550.1			Poecilia mexicana syntaxin-2-like (LOC106932068), transcript variant X2, mRNA	A0A147AU48
324	XM_015005350.1	L + H	**S42_249281**	Poecilia mexicana thyrotropin-releasing hormone (trh), transcript variant X1, mRNA	A0A147B357
	XM_015005351.1			Poecilia mexicana thyrotropin-releasing hormone (trh), transcript variant X2, mRNA	A0A146ZXW2
					A0A146YGA5
					A0A147AVL5
					A0A146SP16
					A0A146VYC6
325	XM_015003153.1	L	**S2711_3408**	Poecilia mexicana transmembrane protein 26 (tmem26), mRNA	Q6ZUK4
					H7BXI3
326	XM_008430474.2	L	**S7384_156**	Poecilia reticulata 1-phosphatidylinositol 4,5-bisphosphate phosphodiesterase delta-4-like (LOC103477381), transcript variant X2, mRNA	Q9BRC7
					H7C237
					H7C281
					C9JAE4
					F2Z3H8
327	XM_008430459.2	B + H	**S368_129091**	Poecilia reticulata 2-aminoethanethiol dioxygenase-like (LOC103477378), transcript variant X3, mRNA	Q7KYR7

328	XM_017301730.1	L	**S183_454422**	Poecilia reticulata ATPase family, AAA domain containing 1 (atad1), transcript variant X2, mRNA	Q8NBU5
329	XM_008403559.2	L	**S1062_14945**	Poecilia reticulata butyrophilin subfamily 1 member A1-like (LOC103461240), mRNA	H7BYC3
					H7C542
					C9JNC3
330	XM_008431539.2	L	**S9941_4782742**	Poecilia reticulata glutamate receptor ionotropic, NMDA 2D-like (LOC103478060), transcript variant X1, mRNA	O15399
	XM_008431538.2			Poecilia reticulata glutamate receptor ionotropic, NMDA 2D-like (LOC103478060), transcript variant X2, mRNA	
	XM_008431540.2			Poecilia reticulata glutamate receptor ionotropic, NMDA 2D-like (LOC103478060), transcript variant X3, mRNA	
331	XM_017306302.1	L	**S9941_4782742**	Poecilia reticulata lysine-specific demethylase 4C-like (LOC103468987), transcript variant X6, mRNA	Q9H3R0
					F8WCN1
					C9J879
					A0A0A0MSR6
332	XM_017301729.1	B	**S9856_2465434**	Poecilia reticulata myeloid-associated differentiation marker-like protein 2 (LOC103481154), mRNA	A6NDP7
333	XM_008424642.2	L	**S2711_3408**	Poecilia reticulata niban-like protein 1 (LOC103473959), mRNA	Q96TA1
334	XM_008407919.2	B	**S9949_1018299**	Poecilia reticulata retinal homeobox protein Rx1 (LOC103464089), mRNA	
335	XM_008417702.1	L	**S1_2497187**	Poecilia reticulata sushi domain containing 1 (susd1), transcript variant X1, mRNA	F8WAQ1
	XM_008417703.1			Poecilia reticulata sushi domain containing 1 (susd1), transcript variant X2, mRNA	H3BLV4
	XM_008417704.1			Poecilia reticulata sushi domain containing 1 (susd1), transcript variant X3, mRNA	H0Y6B2
					H0YCH6
336	XM_008417479.2	L + B	**S9899_1841030**	Poecilia reticulata syntaxin-2 (LOC103469661), transcript variant X1, mRNA	P43155
	XM_008417480.2			Poecilia reticulata syntaxin-2 (LOC103469661), transcript variant X2, mRNA	A0A147AU48
337	XM_008414034.2	L + H	**S42_249281**	Poecilia reticulata thyrotropin releasing hormone (trh), transcript variant X1, mRNA	A0A147B357
	XM_008414035.2			Poecilia reticulata thyrotropin releasing hormone (trh), transcript variant X2, mRNA	A0A146ZXW2
					A0A146YGA5
					A0A147AVL5
					A0A146SP16
					A0A146VYC6
338	XM_008429164.2	L	**S2711_3371**	Poecilia reticulata transmembrane protein 26 (tmem26), mRNA	Q6ZUK4
					H7BXI3

339	XM_017301715.1	L	**S9941_4782742**	Poecilia reticulata urotensin-2 receptor-like (LOC103482075), mRNA	Q9UKP6
340	XM_008416763.2	L	**S174_3611515**	Poecilia reticulata voltage-dependent calcium channel gamma-5 subunit-like (LOC103469220), transcript variant X1, mRNA	Q9UF02
	XM_017306034.1			Poecilia reticulata voltage-dependent calcium channel gamma-5 subunit-like (LOC103469220), transcript variant X2, mRNA	
	XM_008416764.2			Poecilia reticulata voltage-dependent calcium channel gamma-5 subunit-like (LOC103469220), transcript variant X3, mRNA	
	XM_017306035.1			Poecilia reticulata voltage-dependent calcium channel gamma-5 subunit-like (LOC103469220), transcript variant X4, mRNA	
	XM_017306036.1			Poecilia reticulata voltage-dependent calcium channel gamma-5 subunit-like (LOC103469220), transcript variant X5, mRNA	
342	XM_017306950.1	L	**S703_83370**	Poecilia reticulata WAS/WASL-interacting protein family member 3-like (LOC108166601), transcript variant X2, mRNA	A6NGB9
	XM_017306951.1			Poecilia reticulata WAS/WASL-interacting protein family member 3-like (LOC108166601), transcript variant X3, mRNA	A0A0A0MSG0
	XM_017306952.1			Poecilia reticulata WAS/WASL-interacting protein family member 3-like (LOC108166601), transcript variant X4, mRNA	
343	NM_001133889.1	L	**S9929_1038014**	Pongo abelii KH RNA binding domain containing, signal transduction associated 3 (KHDRBS3), mRNA	
344	NM_001134211.1	B	**S3317_962**	Pongo abelii THAP domain containing 10 (THAP10), mRNA	Q9P2Z0
					H0YN95
345	AF217957.1	B	**S3317_962**	Populus tremuloides cinnamyl alcohol dehydrogenase mRNA, complete cds	
346	XM_021963250.1	L	**S9866_174755**	Prunus avium probable serine/threonine-protein kinase PIX7 (LOC110760898), mRNA	E9PIX7
347	XM_020436610.1	B	**S771_52821**	Pseudomyrmex gracilis kinesin heavy chain (LOC109858903), mRNA	Q12840
					P33176
					O60282
348	XM_017703180.1	L	**S9906_191627**	Pygocentrus nattereri actin related protein 2/3 complex subunit 1A (arpc1a), mRNA	Q92747
					E9PF58
					Q75MY0
349	BC099116.1	L	**S9929_1038014**	Rattus norvegicus M-phase phosphoprotein 8, mRNA (cDNA clone MGC:116256 IMAGE:7462184), complete cds	Q99549
350	NM_001024890.1	B	**S3317_962**	Rattus norvegicus PIH1 domain containing 3 (Pih1d3), mRNA	Q9NQM4
351	NM_001014173.1	L	**S9929_1038014**	Rattus norvegicus ribulose-5-phosphate-3-epimerase (Rpe), mRNA	Q96AT9
352	NM_001033903.1	L	**S885_89904**	Rattus norvegicus ribosomal RNA adenine dimethylase domain containing 1 (Rrnad1), mRNA	Q96FB5
					H0YBL9
					E5RHI7
					E5RI42

					E5RJL6
					E5RIL6
353	XM_017856932.1	L	**S9866_174755**	Rhinopithecus bieti protein tyrosine phosphatase, receptor type Z1 (PTPRZ1), mRNA	P23471
354	XM_010367171.1	L	**S178_234532**	Rhinopithecus roxellana KH-type splicing regulatory protein (KHSRP), transcript variant X1, mRNA	A0A087WTP3
	XM_010367172.1			Rhinopithecus roxellana KH-type splicing regulatory protein (KHSRP), transcript variant X2, mRNA	M0R3J3
	XM_010367174.1			Rhinopithecus roxellana KH-type splicing regulatory protein (KHSRP), transcript variant X3, mRNA	Q92945
355	NM_001168153.1	L	**S1100_29850**	Saccoglossus kowalevskii p75 neurotrophin receptor-like protein (LOC100313698), mRNA	P08138
					Q00994
					Q9Y5V3
					P04629
					Q9Y4K3
					P01138
					O00327
					Q99523
356	XM_010349198.1	H	**S217_136844**	Saimiri boliviensis boliviensis aspartate beta-hydroxylase domain containing 2 (ASPHD2), mRNA	A0A024R1D0
					Q6ICH7
357	XM_003941777.2	B	**S3317_962**	Saimiri boliviensis boliviensis ELAV like neuron-specific RNA binding protein 3 (ELAVL3), transcript variant X1, mRNA	Q14576
	XM_003941776.2			Saimiri boliviensis boliviensis ELAV like neuron-specific RNA binding protein 3 (ELAVL3), transcript variant X2, mRNA	Q96J71
					K7EPB5
					Q9H024
					L8E8Z0
358	XR_743666.1	B	**S3317_962**	Saimiri boliviensis boliviensis proline-rich receptor-like protein kinase PERK2 (LOC104649891), misc_RNA	P55085
359	XM_014160280.1	L + B	**S9899_1841030**	Salmo salar syntaxin 2 (stx2), mRNA	P43155
					A0A147AU48
360	EU025709.1	L	**S10156_378391**	Salmo salar chromatin modifying protein 2a (Chmp2a), male-specific lethal-1-like protein (Hampin), gastric inhibitory polypeptide (Gip), EAP30 subunit of ELL complex b (Eap30b), and nuclear domain 10 protein 52b (Ndp52b) genes, complete cds; BAC05032 (BAC05032) pseudogene, partial sequence; HoxB8bb (HoxB8bb) pseudogene, complete sequence; homeobox protein HoxB6bb (HoxB6bb) and homeobox protein HoxB5bb (HoxB5bb) genes, complete cds; and HoxB3bb and HoxB1bb pseudogenes, complete sequence	O43633

					A0A024R4S0
					M0R1L7
					M0QXX8
					M0R1T5
361	NM_001173625.1	B	**S8943_473**	Salmo salar procollagen-proline, 2-oxoglutarate 4-dioxygenase (proline 4-hydroxylase), alpha polypeptide I (p4ha1), mRNA	P13674
					Q9UKV8
					P02671
					P42858
					Q5VSQ6
362	XM_003769128.2	L	**S885_89904**	Sarcophilus harrisii 24-hydroxycholesterol 7-alpha-hydroxylase (LOC100919523), mRNA	Q9NYL5
					A0A087WTD2
363	XM_022756632.1	L + B	**S9899_1841030**	Seriola dumerili syntaxin-2-like (LOC111230065), transcript variant X1, mRNA	P43155
	XM_022756640.1			Seriola dumerili syntaxin-2-like (LOC111230065), transcript variant X2, mRNA	A0A147AU48
	XM_022756649.1			Seriola dumerili syntaxin-2-like (LOC111230065), transcript variant X3, mRNA	
364	XM_023393890.1	L + B	**S9899_1841030**	Seriola lalandi dorsalis syntaxin 2 (stx2), transcript variant X1, mRNA	P43155
	XM_023393891.1			Seriola lalandi dorsalis syntaxin 2 (stx2), transcript variant X2, mRNA	A0A147AU48
365	XM_016263825.1	L	**S178_234532**	Sinocyclocheilus grahami 5-methylcytosine rRNA methyltransferase NSUN4-like (LOC107577791), mRNA	Q96CB9
					M0R1K5
					Q6ZRQ1
					A0A087X0V6
					A0A087WT36
					A0A087WUV3
366	XM_016288381.1	H	**S9874_1914529**	Sinocyclocheilus grahami immunoglobulin superfamily member 3-like (LOC107597363), transcript variant X3, mRNA	O75054
367	XM_016259949.1	H	**S1043_75350**	Sinocyclocheilus grahami putative nuclease HARBI1 (LOC107573514), mRNA	E9PK24
					E9PQI1
368	XM_008276734.1	L + B	**S9899_1841030**	Stegastes partitus syntaxin 2 (stx2), mRNA	P43155
					A0A147AU48
369	XM_008304105.1	B	**S8943_473**	Stegastes partitus transforming protein RhoA-like (LOC103374076), mRNA	P61586

370	XM_009688078.1	L	**S9853_2824879**	Struthio camelus australis ubiquitin specific peptidase 9, X-linked (USP9X), transcript variant X1, mRNA	Q93008
	XM_009688085.1			Struthio camelus australis ubiquitin specific peptidase 9, X-linked (USP9X), transcript variant X2, mRNA	D3DWB6
					Q6P468
					Q59EZ5
					Q86X58
371	AJ459419.1	L	**S124_317291**	Takifugu rubripes cacng5 gene, prkca gene and putative LINE-like retrotransposon, cosmids C012B17 and C123B01	P17252
372	XM_009984321.1	B	**S771_52821**	Tauraco erythrolophus Wiskott-Aldrich syndrome-like (WASL), transcript variant X2, mRNA	O00401
373	JX459927.1	L	**S9929_1038014**	Thunnus thynnus kisspeptin 2 (Kiss2) mRNA, complete cds	Q15726
					A0A0D9SES6
374	XM_026391212.1	L	**S9853_2824879**	Urocitellus parryii zinc finger protein 536 (Znf536), mRNA	O15090
					K7EKT4
					A7E228
					K7EQN6
					K7EJP8
375	XM_025983505.1	B	**S3317_962**	Vulpes vulpes ELAV like RNA binding protein 3 (ELAVL3), transcript variant X1, mRNA	Q14576
	XM_025983506.1			Vulpes vulpes ELAV like RNA binding protein 3 (ELAVL3), transcript variant X2, mRNA	Q96J71
	XM_025983507.1			Vulpes vulpes ELAV like RNA binding protein 3 (ELAVL3), transcript variant X3, mRNA	K7EPB5
					Q9H024
					L8E8Z0
376	XM_018235011.1	L	**S9929_1038014**	Xenopus laevis zinc finger CCCH-type with G patch domain-containing protein (LOC108700950), mRNA	Q8N5A5
					X6RGY1
377	XM_023328054.1	B + H	**S368_129091**	Xiphophorus maculatus 2-aminoethanethiol dioxygenase-like (LOC102220352), mRNA	Q96SZ5
378	XM_023337701.1	L	**S703_83370**	Xiphophorus maculatus AP2-associated protein kinase 1-like (LOC102221429), transcript variant X1, mRNA	Q2M2I8
	XM_023337702.1			Xiphophorus maculatus AP2-associated protein kinase 1-like (LOC102221429), transcript variant X2, mRNA	E9PG46
					D6W5G0
					A0A096LNZ0
					A0A096LP25
					A0A096LP60

379	XM_023343922.1	H	**S9873_366940**	Xiphophorus maculatus checkpoint with forkhead and ring finger domains (chfr), transcript variant X2, mRNA	Q96EP1
	XM_023343922.1			Xiphophorus maculatus checkpoint with forkhead and ring finger domains (chfr), transcript variant X2, mRNA	F5GWH4
					A0A096P6K8
					F5H829
					F5H375
					A0A087X0W6
					A0A087WUN4
					F5H5P5
					U3KPU9
380	XM_023332533.1	L	**S9941_4782742**	Xiphophorus maculatus CUB and Sushi multiple domains 1 (csmd1), transcript variant X1, mRNA	Q96PZ7
	XM_023332534.1			Xiphophorus maculatus CUB and Sushi multiple domains 1 (csmd1), transcript variant X2, mRNA	E5RIG2
	XM_023332535.1			Xiphophorus maculatus CUB and Sushi multiple domains 1 (csmd1), transcript variant X3, mRNA	F8W9C3
	XM_023332537.1			Xiphophorus maculatus CUB and Sushi multiple domains 1 (csmd1), transcript variant X4, mRNA	F5GZ18
					H7BXU2
381	XM_005809517.3	L	**S2711_3408**	Xiphophorus maculatus cysteinyl-tRNA synthetase (cars), transcript variant X1, mRNA	P49589
	XM_023332812.1			Xiphophorus maculatus cysteinyl-tRNA synthetase (cars), transcript variant X2, mRNA	B4DKY1
	XM_023332813.1			Xiphophorus maculatus cysteinyl-tRNA synthetase (cars), transcript variant X3, mRNA	A0A024RCG3
					B4DPV7
					A8MVQ3
					A0A087WWV1
					A8K067
					F5H579
					H0YFF0
					H0YFV1
					F5H623
					E9PRS8
					H0YGF2
					E9PLP0

					C9JLN0
382	XM_023340137.1	L	**S9941_4782742**	Xiphophorus maculatus diencephalon/mesencephalon homeobox protein 1-B-like (LOC102237364), transcript variant X1, mRNA	Q8NFW5
	XM_005803931.2			Xiphophorus maculatus diencephalon/mesencephalon homeobox protein 1-B-like (LOC102237364), transcript variant X2, mRNA	
383	XM_023332697.1	L	**S2711_3408**	Xiphophorus maculatus immunoglobulin superfamily DCC subclass member 4 (LOC102231531), transcript variant X1, mRNA	Q8TDY8
	XM_023332698.1			Xiphophorus maculatus immunoglobulin superfamily DCC subclass member 4 (LOC102231531), transcript variant X2, mRNA	
384	XM_023342989.1	L	**S2711_3408**	Xiphophorus maculatus inositol hexakisphosphate and diphosphoinositol-pentakisphosphate kinase 2-like (LOC102227875), transcript variant X13, mRNA	O43314
					A0A087WZV0
					D6RBU4
					H0Y9S9
385	XM_023334057.1	L	**S2711_3408**	Xiphophorus maculatus interleukin 1 receptor associated kinase 1 (irak1), transcript variant X1, mRNA	P51617
	XM_023334063.1			Xiphophorus maculatus interleukin 1 receptor associated kinase 1 (irak1), transcript variant X2, mRNA	
	XM_023334065.1			Xiphophorus maculatus interleukin 1 receptor associated kinase 1 (irak1), transcript variant X3, mRNA	
386	XM_023333689.1	L	**S9941_4782742**	Xiphophorus maculatus lysine-specific demethylase 4C-like (LOC102223593), transcript variant X4, mRNA	Q9H3R0
					F8WCN1
					C9J879
					A0A0A0MSR6
387	XM_023328262.1	L	**S703_83370**	Xiphophorus maculatus myotilin (myot), transcript variant X5, mRNA	Q9UBF9
					A0A0S2Z4Y0
					A0A0C4DFM5
					B4DT68
					A0A0S2Z522
388	XM_023338326.1	B	**S53_432183**	Xiphophorus maculatus NACHT, LRR and PYD domains-containing protein 12-like (LOC102228242), transcript variant X1, mRNA	P59046
	XM_023338327.1			Xiphophorus maculatus NACHT, LRR and PYD domains-containing protein 12-like (LOC102228242), transcript variant X2, mRNA	A0A0C4DFY3
	XM_014474269.2			Xiphophorus maculatus NACHT, LRR and PYD domains-containing protein 12-like (LOC102228242), transcript variant X3, mRNA	A0A0C4DH17
389	XM_023325262.1	L	**S2711_3408**	Xiphophorus maculatus PDX1 C-terminal inhibiting factor 1 (pcif1), transcript variant X1, mRNA	Q9H4Z3
	XM_005812943.3			Xiphophorus maculatus PDX1 C-terminal inhibiting factor 1 (pcif1), transcript variant X2, mRNA	A0A087WWZ2

	XM_023325263.1			Xiphophorus maculatus PDX1 C-terminal inhibiting factor 1 (pcif1), transcript variant X3, mRNA	
	XM_023325264.1			Xiphophorus maculatus PDX1 C-terminal inhibiting factor 1 (pcif1), transcript variant X4, mRNA	
	XM_023325265.1			Xiphophorus maculatus PDX1 C-terminal inhibiting factor 1 (pcif1), transcript variant X5, mRNA	
	XM_023325266.1			Xiphophorus maculatus PDX1 C-terminal inhibiting factor 1 (pcif1), transcript variant X6, mRNA	
390	XM_005804340.3	L	**S703_83370**	Xiphophorus maculatus phosphatidylinositol transfer protein beta (pitpnb), transcript variant X2, mRNA	P48739
					A0A0A0MSW4
					B2R7P6
					B3KYB6
					B3KYB7
391	XM_023343353.1	H	**S9873_366940**	Xiphophorus maculatus pulmonary surfactant-associated protein D-like (LOC111610285), mRNA	P35247
					Q5T0M2
					G5CCM8
392	XM_023347384.1	L	**S703_83370**	Xiphophorus maculatus putative deoxyribonuclease tatdn3 (LOC102218828), transcript variant X1, mRNA	Q17R31
	XM_023347385.1			Xiphophorus maculatus putative deoxyribonuclease tatdn3 (LOC102218828), transcript variant X2, mRNA	E9PP81
	XM_023347386.1			Xiphophorus maculatus putative deoxyribonuclease tatdn3 (LOC102218828), transcript variant X3, mRNA	E9PRA1
	XM_023347387.1			Xiphophorus maculatus putative deoxyribonuclease tatdn3 (LOC102218828), transcript variant X4, mRNA	E9PQP8
	XM_023347389.1			Xiphophorus maculatus putative deoxyribonuclease tatdn3 (LOC102218828), transcript variant X6, mRNA	H0YCC7
					H0YD82
					E9PPF0
					A0A1S7LLX1
					U3KQ29
					H0YCN0
393	XM_023330764.1	L	**S703_83370**	Xiphophorus maculatus RNA binding motif protein 12B (rbm12b), transcript variant X2, mRNA	Q8IXT5
					B9ZVT1
					E5RJV8
					E5RHG1
					E5RJW8

					E5RJ83
394	XM_023350157.1	L	**S174_3611515**	Xiphophorus maculatus round spermatid basic protein 1-like (LOC102219208), mRNA	Q5VWQ0
					Q6PCB5
					C9JM20
					H7C2D3
					A0A0C4DH79
395	XM_023351494.1	B + H	**S368_129091**	Xiphophorus maculatus serine/threonine-protein kinase PAK 4 (LOC102225392), transcript variant X1, mRNA	A0A024R0J1
	XM_023351495.1			Xiphophorus maculatus serine/threonine-protein kinase PAK 4 (LOC102225392), transcript variant X2, mRNA	A0A024R0L8
	XR_002754356.1			Xiphophorus maculatus serine/threonine-protein kinase PAK 4 (LOC102225392), transcript variant X3, misc_RNA	M0R1R1
					M0R0L9
					M0R2X4
					M0R3G6
					B4DUG0
396	XM_023350876.1	L	**S703_83370**	Xiphophorus maculatus solute carrier family 33 member 1 (slc33a1), transcript variant X1, mRNA	O00400
	XM_023350877.1			Xiphophorus maculatus solute carrier family 33 member 1 (slc33a1), transcript variant X2, mRNA	H7C577
					H7C562
					H7C532
					A0A2R8YF57
					A0A2R8Y359
					A0A2R8Y5I5
397	XM_023343215.1	L + B	**S9899_1841030**	Xiphophorus maculatus syntaxin-2 (LOC102236004), transcript variant X1, mRNA	P43155
	XM_005806921.2			Xiphophorus maculatus syntaxin-2 (LOC102236004), transcript variant X2, mRNA	A0A147AU48
	XM_023343217.1			Xiphophorus maculatus syntaxin-2 (LOC102236004), transcript variant X3, mRNA	
	XM_023343218.1			Xiphophorus maculatus syntaxin-2 (LOC102236004), transcript variant X4, mRNA	
398	XM_005805658.2	L + H	**S42_249281**	Xiphophorus maculatus thyrotropin releasing hormone (trh), transcript variant X1, mRNA	A0A147B357
	XM_005805659.2			Xiphophorus maculatus thyrotropin releasing hormone (trh), transcript variant X2, mRNA	A0A146ZXW2
					A0A146YGA5
					A0A147AVL5

					A0A146SP16
					A0A146VYC6
399	XM_023348925.1	L	**S2711_3408**	Xiphophorus maculatus trinucleotide repeat containing 18 (tnrc18), transcript variant X1, mRNA	O15417
	XM_014471422.2			Xiphophorus maculatus trinucleotide repeat containing 18 (tnrc18), transcript variant X2, mRNA	H9KVB4
	XM_023348927.1			Xiphophorus maculatus trinucleotide repeat containing 18 (tnrc18), transcript variant X3, mRNA	H7C0N9
					H7BXS9
					H7C3U5
					A8MTZ4
					H7C177
					C9J9K1
					A3KMH2
400	XM_023348487.1	L	**S703_83370**	Xiphophorus maculatus ubiquitin carboxyl-terminal hydrolase 43-like (LOC102235173), transcript variant X1, mRNA	Q70EL4
	XM_023348488.1			Xiphophorus maculatus ubiquitin carboxyl-terminal hydrolase 43-like (LOC102235173), transcript variant X2, mRNA	V9GZ20
					V9GXZ5
401	XM_023348688.1	L	**S174_3611515**	Xiphophorus maculatus voltage-dependent calcium channel gamma-5 subunit-like (LOC102227037), transcript variant X1, mRNA	Q9UF02
	XM_005808875.2			Xiphophorus maculatus voltage-dependent calcium channel gamma-5 subunit-like (LOC102227037), transcript variant X2, mRNA	
402	XM_023348828.1	L	**S192_63422**	Xiphophorus maculatus zinc finger protein 652 (znf652), transcript variant X1, mRNA	Q9Y2D9
	XM_023348829.1			Xiphophorus maculatus zinc finger protein 652 (znf652), transcript variant X2, mRNA	D6RF85
	XM_023348830.1			Xiphophorus maculatus zinc finger protein 652 (znf652), transcript variant X3, mRNA	A8K9F2
	XM_023348831.1			Xiphophorus maculatus zinc finger protein 652 (znf652), transcript variant X4, mRNA	
	XM_023348832.1			Xiphophorus maculatus zinc finger protein 652 (znf652), transcript variant X5, mRNA	
403	NM_001086195.1	L	**S9929_1038014**	Xenopus laevis Actin, alpha cardiac muscle 2-like (MGC53823), mRNA	P35609
404	NM_001094741.1	B	**S8943_473**	Xenopus laevis aquaporin 1 (Colton blood group) S homeolog (aqp1.S), mRNA	P29972
405	NM_001092374.1	H	**S9889_2454843**	Xenopus laevis ArfGAP with coiled-coil, ankyrin repeat and PH domains 1 S homeolog (acap1.S), mRNA	Q15027
					I3L0K9
					I3L268
406	NM_001092784.1	L	**S9929_1038014**	Xenopus laevis coiled-coil domain containing 28B L homeolog (ccdc28b.L), mRNA	Q9BUN5

					E9PM81<br>E9PM03
407	NM_001093549.1	L	**S885_89904**	Xenopus laevis cytochrome b-245 alpha polypeptide L homeolog (cyba.L), mRNA	P13498<br>H3BNP7<br>H3BR52<br>H3BPX1<br>H3BT77
408	NM_001095540.1	L	**S9929_1038014**	Xenopus laevis forkhead box I2 S homeolog (foxi2.S), mRNA	C4IXT1<br>Q6ZQN5
409	NM_001096224.1	L	**S9866_809619**	Xenopus laevis sperm-tail PG-rich repeat containing 1 L homeolog (stpg1.L), mRNA	Q5TH74<br>Q5TH77<br>H0Y4J3
410	NM_001097722.1	L	**S9853_2824879**	Xenopus laevis TruB pseudouridine (psi) synthase family member 2 L homeolog (trub2.L), mRNA	O95900<br>A0A024R886
411	NM_001093137.1	L	**S9966_92994**	Xenopus laevis zinc finger protein 484 L homeolog (znf484.L), mRNA	Q5JVG2
412	NM_001095068.1	L	**S9929_1038014**	Xenopus laevis zinc finger, CCCH-type with G-patch domain L homeolog (zgpat.L), mRNA	Q8N5A5
413	NM_213666.2	L	**S9853_2824879**	Xenopus tropicalis 6-phosphofructo-2-kinase/fructose-2,6-biphosphatase 4 (pfkfb4), mRNA	Q16877<br>Q66S35<br>C9K0D8<br>C9JX77<br>C9JJ23<br>B2R6L2<br>F8WC12<br>F8WDY1

N = consecutive number of gene listed. They are presented as a cluster when a gene was containing more than one variant.
L = Lositan; B = Bayenv2; H = Hierarchical Island Model

Table S4. Enrichment analysis: functional clusters. The table explains the associations between groups of targeted genes (Uniprot accessions) and the functional terms identified in the significant 16 clusters identified by DAVID 6.7 (Huang et al., 2009).

Annotation Cluster 1	**Function: Metal-thiolate cluster (Antioxydant) (ES = 20.55)**				
Category	Term	Count	%	PValue	Genes
UP_KEYWORDS	Metal-thiolate cluster	14	3.24074074	1.07E-21	A0A024R6R7, A1L3X4, H3BR34, P07438, P04733, H3BQX6, P04732, H3BSS0, P04731, Q93083, P13640, H3BRY8, P25713, P80294, H3BTG5, H3BSP9, P80297, P0DM35, P47944, P02795, Q8N339, U3KQD7
INTERPRO	IPR023587:Metallothionein domain, vertebrate	14	3.24074074	3.61E-21	A0A024R6R7, A1L3X4, H3BR34, P07438, P04733, H3BQX6, P04732, H3BSS0, P04731, Q93083, P13640, H3BRY8, P25713, P80294, H3BTG5, H3BSP9, P80297, P0DM35, P47944, P02795, Q8N339, U3KQD7
INTERPRO	IPR017854:Metallothionein domain	14	3.24074074	3.61E-21	A0A024R6R7, A1L3X4, H3BR34, P07438, P04733, H3BQX6, P04732, H3BSS0, P04731, Q93083, P13640, H3BRY8, P25713, P80294, H3BTG5, H3BSP9, P80297, P0DM35, P47944, P02795, Q8N339, U3KQD7
INTERPRO	IPR003019:Metallothionein superfamily, eukaryotic	14	3.24074074	3.61E-21	A0A024R6R7, A1L3X4, H3BR34, P07438, P04733, H3BQX6, P04732, H3BSS0, P04731, Q93083, P13640, H3BRY8, P25713, P80294, H3BTG5, H3BSP9, P80297, P0DM35, P47944, P02795, Q8N339, U3KQD7
INTERPRO	IPR000006:Metallothionein, vertebrate	14	3.24074074	3.61E-21	A0A024R6R7, A1L3X4, H3BR34, P07438, P04733, H3BQX6, P04732, H3BSS0, P04731, Q93083, P13640, H3BRY8, P25713, P80294, H3BTG5, H3BSP9, P80297, P0DM35, P47944, P02795, Q8N339, U3KQD7

Annotation Cluster 2	**Function: Metal Binding proteins (ES = 14.88)**				
Category	Term	Count	%	PValue	Genes
INTERPRO	IPR018064:Metallothionein, vertebrate, metal binding site	11	2.5462963	2.81E-16	A0A024R6R7, H3BR34, P07438, H3BQX6, P04733, H3BSS0, P04732, P04731, Q93083, P13640, P80294, P25713, H3BRY8, H3BTG5, P80297, H3BSP9, P02795, U3KQD7, Q8N339
UP_SEQ_FEATURE	region of interest:Beta	11	2.5462963	2.85E-15	A0A024R6R7, H3BR34, P07438, H3BQX6, P04733, H3BSS0, P04732, P04731, Q93083, P13640, P80294, P25713, H3BRY8, H3BTG5, P80297, H3BSP9, P02795, U3KQD7, Q8N339
UP_SEQ_FEATURE	region of interest:Alpha	11	2.5462963	2.85E-15	A0A024R6R7, H3BR34, P07438, H3BQX6, P04733, H3BSS0, P04732, P04731, Q93083, P13640, P80294, P25713, H3BRY8, H3BTG5, P80297, H3BSP9, P02795, U3KQD7, Q8N339

Annotation Cluster 3	**Function: Nitrogen metabolism (ES = 12.59)**				
Category	Term	Count	%	PValue	Genes
GOTERM_MF_DIRECT	GO:0004089~carbonate dehydratase activity	11	2.5462963	2.82E-14	P23280, P00915, Q9ULX7, Q16790, P07451, Q8N1Q1, P35219, P43166, O43570, P00918, P22748
INTERPRO	IPR023561:Carbonic anhydrase, alpha-class	11	2.5462963	1.85E-13	P23280, P00915, Q9ULX7, Q16790, P07451, Q8N1Q1, P35219, P43166, O43570, P00918, P22748
KEGG_PATHWAY	hsa00910:Nitrogen metabolism	11	2.5462963	3.36E-12	P23280, P00915, Q9ULX7, Q16790, P07451, Q8N1Q1, P35219, P43166, O43570, P00918, P22748

Annotation Cluster 4	**Function: Fatty acid metabolism (ES = 4.48)**				
Category	Term	Count	%	PValue	Genes
INTERPRO	IPR000463:Cytosolic fatty-acid binding	7	1.62037037	1.16E-06	Q01469, P51161, P05413, O15540, P15090, P12104, P07148
INTERPRO	IPR012674:Calycin	7	1.62037037	1.50E-04	Q01469, P51161, P05413, O15540, P15090, P12104, P07148
INTERPRO	IPR011038:Calycin-like	7	1.62037037	2.02E-04	Q01469, P51161, P05413, O15540, P15090, P12104, P07148

Annotation Cluster 5	**Function: mechanisms of signal transduction via tyrosine kinases (ES = 4.19)**				
Category	Term	Count	%	PValue	Genes
INTERPRO	IPR000494:EGF receptor, L domain	5	1.15740741	7.95E-06	P04626, P21860, P06213, Q15303, P00533, A0A024RB84
INTERPRO	IPR006211:Furin-like cysteine-rich domain	5	1.15740741	7.95E-06	P04626, P21860, P06213, Q15303, P00533, A0A024RB84
GOTERM_MF_DIRECT	GO:0004716~receptor signaling protein tyrosine kinase activity	5	1.15740741	4.82E-05	P04626, P21860, P06213, Q15303, P00533, A0A024RB84
INTERPRO	IPR006212:Furin-like repeat	5	1.15740741	5.72E-04	P04626, P21860, P06213, Q15303, P00533, A0A024RB84
SMART	SM00261:FU	5	1.15740741	6.55E-04	P04626, P21860, P06213, Q15303, P00533, A0A024RB84

Annotation Cluster 6	**Function: Tyrosin kinase catalitic asctivity (ES = 2.38)**				
Category	Term	Count	%	PValue	Genes
INTERPRO	IPR020635:Tyrosine-protein kinase, catalytic domain	8	1.85185185	0.00281881	P06239, Q5T244, Q5T245, A0A024R906, Q15303, P04626, Q5T241, H0Y570, Q16832, P04629, P21860, P06213, P00533, A0A024RB84
SMART	SM00219:TyrKc	8	1.85185185	0.00340702	P06239, Q5T244, Q5T245, A0A024R906, Q15303, P04626, Q5T241, H0Y570, Q16832, P04629, P21860, P06213, P00533, A0A024RB84
UP_KEYWORDS	Tyrosine-protein kinase	8	1.85185185	0.007728	P06239, Q5T244, Q5T245, A0A024R906, Q15303, P04626, Q5T241, H0Y570, Q16832, P04629, P21860, P06213, P00533, A0A024RB84

Annotation Cluster 7	**Function: subcellular (cytoskeleton) localization (ES = 1.62)**				
Category	Term	Count	%	PValue	Genes
INTERPRO	IPR010473:Diaphanous GTPase-binding	3	0.69444444	0.01596229	K7EJE6, F8VYL1, Q96PY5, Q8IVF7, K7EMY8, K7ERL1, A0A0A0MR62, F8W1F5, K7EK60, O95466, C9IZY8
INTERPRO	IPR010472:Diaphanous FH3	3	0.69444444	0.01596229	K7EJE6, F8VYL1, Q96PY5, Q8IVF7, K7EMY8, K7ERL1, A0A0A0MR62, F8W1F5, K7EK60, O95466, C9IZY8
SMART	SM01140:SM01140	3	0.69444444	0.01714181	K7EJE6, F8VYL1, Q96PY5, Q8IVF7, K7EMY8, K7ERL1, A0A0A0MR62, F8W1F5, K7EK60, O95466, C9IZY8
SMART	SM01139:SM01139	3	0.69444444	0.01714181	K7EJE6, F8VYL1, Q96PY5, Q8IVF7, K7EMY8, K7ERL1, A0A0A0MR62, F8W1F5, K7EK60, O95466, C9IZY8
UP_SEQ_FEATURE	domain:GBD/FH3	3	0.69444444	0.02000358	K7EJE6, F8VYL1, Q96PY5, Q8IVF7, K7EMY8, K7ERL1, A0A0A0MR62, F8W1F5, K7EK60, O95466, C9IZY8
INTERPRO	IPR014768:GTPase-binding/formin homology 3	3	0.69444444	0.02368352	K7EJE6, F8VYL1, Q96PY5, Q8IVF7, K7EMY8, K7ERL1, A0A0A0MR62, F8W1F5, K7EK60, O95466, C9IZY8
UP_SEQ_FEATURE	domain:FH2	3	0.69444444	0.03620894	K7EJE6, F8VYL1, Q96PY5, Q8IVF7, K7EMY8, K7ERL1, A0A0A0MR62, F8W1F5, K7EK60, O95466, C9IZY8
INTERPRO	IPR015425:Actin-binding FH2	3	0.69444444	0.04265806	K7EJE6, F8VYL1, Q96PY5, Q8IVF7, K7EMY8, K7ERL1, A0A0A0MR62, F8W1F5, K7EK60, O95466, C9IZY8
SMART	SM00498:FH2	3	0.69444444	0.04566941	K7EJE6, F8VYL1, Q96PY5, Q8IVF7, K7EMY8, K7ERL1, A0A0A0MR62, F8W1F5, K7EK60, O95466, C9IZY8

Annotation Cluster 8	**Function: steroid/arachidonic acid metabolism (ES = 1.59)**				
Category	Term	Count	%	PValue	Genes
GOTERM_MF_DIRECT	GO:0008392~arachidonic acid epoxygenase activity	4	0.92592593	0.00424133	Q9UQ59, S5RV29, G3V188, S5RXA4, P33260, P33261, S5RG22, P10632, P11712, S5RV20, Q9UEH3, A0A087X125, Q2XN56, S5R8H1, S5R8G8, E9PLI9, B7Z1F5, E9PIW6
GOTERM_BP_DIRECT	GO:0019373~epoxygenase P450 pathway	4	0.92592593	0.00728785	Q9UQ59, S5RV29, G3V188, S5RXA4, P33260, P33261, S5RG22, P10632, P11712, S5RV20, Q9UEH3, A0A087X125, Q2XN56, S5R8H1, S5R8G8, E9PLI9, B7Z1F5, E9PIW6
GAD_DISEASE	warfarin sensitivity	4	0.92592593	0.00969021	Q9UQ59, S5RV29, G3V188, S5RXA4, P33260, P33261, S5RG22, P10632, P11712, S5RV20, Q9UEH3, A0A087X125, Q2XN56, S5R8H1, S5R8G8, E9PLI9, B7Z1F5, E9PIW6
GOTERM_MF_DIRECT	GO:0008395~steroid hydroxylase activity	4	0.92592593	0.0201886	Q9UQ59, S5RV29, G3V188, S5RXA4, P33260, P33261, S5RG22, P10632, P11712, S5RV20, Q9UEH3, A0A087X125, Q2XN56, S5R8H1, S5R8G8, E9PLI9, B7Z1F5, E9PIW6
GOTERM_MF_DIRECT	GO:0019825~oxygen binding	4	0.92592593	0.08944502	Q9UQ59, S5RV29, G3V188, S5RXA4, P33260, P33261, S5RG22, P10632, P11712, S5RV20, Q9UEH3, A0A087X125, Q2XN56, S5R8H1, S5R8G8, E9PLI9, B7Z1F5, E9PIW6
INTERPRO	IPR002401:Cytochrome P450, E-class, group I	4	0.92592593	0.09930195	Q9UQ59, S5RV29, G3V188, S5RXA4, P33260, P33261, S5RG22, P10632, P11712, S5RV20, Q9UEH3, A0A087X125, Q2XN56, S5R8H1, S5R8G8, E9PLI9, B7Z1F5, E9PIW6
INTERPRO	IPR017972:Cytochrome P450, conserved site	4	0.92592593	0.13791879	Q9UQ59, S5RV29, G3V188, S5RXA4, P33260, P33261, S5RG22, P10632, P11712, S5RV20, Q9UEH3, A0A087X125, Q2XN56, S5R8H1, S5R8G8, E9PLI9, B7Z1F5, E9PIW6

Annotation Cluster 9	**Function: Drugs/xenobiotics metabolism (ES = 1.34)**				
Category	Term	Count	%	PValue	Genes
COG_ONTOLOGY	Secondary metabolites biosynthesis, transport, and catabolism	5	1.15740741	0.02284499	Q9UQ59, Q9NYL5, S5RV29, G3V188, S5RXA4, P33260, P33261, S5RG22, P10632, P11712, S5RV20, Q9UEH3, A0A087X125, A0A087WTD2, Q2XN56, S5R8H1, S5R8G8, E9PLI9, B7Z1F5, E9PIW6
GOTERM_MF_DIRECT	GO:0016705~oxidoreductase activity, acting on paired donors, with incorporation or reduction of molecular oxygen	5	1.15740741	0.03956108	Q9UQ59, Q9NYL5, S5RV29, G3V188, S5RXA4, P33260, P33261, S5RG22, P10632, P11712, S5RV20, Q9UEH3, A0A087X125, A0A087WTD2, Q2XN56, S5R8H1, S5R8G8, E9PLI9, B7Z1F5, E9PIW6
GOTERM_MF_DIRECT	GO:0004497~monooxygenase activity	5	1.15740741	0.04176411	Q9UQ59, Q9NYL5, S5RV29, G3V188, S5RXA4, P33260, P33261, S5RG22, P10632, P11712, S5RV20, Q9UEH3, A0A087X125, A0A087WTD2, Q2XN56, S5R8H1, S5R8G8, E9PLI9, B7Z1F5, E9PIW6
INTERPRO	IPR001128:Cytochrome P450	5	1.15740741	0.04352862	Q9UQ59, Q9NYL5, S5RV29, G3V188, S5RXA4, P33260, P33261, S5RG22, P10632, P11712, S5RV20, Q9UEH3, A0A087X125, A0A087WTD2, Q2XN56, S5R8H1, S5R8G8, E9PLI9, B7Z1F5, E9PIW6
UP_KEYWORDS	Monooxygenase	5	1.15740741	0.10290758	Q9UQ59, Q9NYL5, S5RV29, G3V188, S5RXA4, P33260, P33261, S5RG22, P10632, P11712, S5RV20, Q9UEH3, A0A087X125, A0A087WTD2, Q2XN56, S5R8H1, S5R8G8, E9PLI9, B7Z1F5, E9PIW6

Annotation Cluster 10	**Function: DNA expression regulation (ES = 1.29)**				
Category	Term	Count	%	PValue	Genes
GOTERM_BP_DIRECT	GO:0043982~histone H4-K8 acetylation	3	0.69444444	0.04972924	D6RAS5, D6RFK0, D6RCS1, Q6IE81, D6RFZ5, D6RC05, D6RE74, D6RGE7, O95251, D6RBB3, Q9BVI0
GOTERM_BP_DIRECT	GO:0043981~histone H4-K5 acetylation	3	0.69444444	0.04972924	D6RAS5, D6RFK0, D6RCS1, Q6IE81, D6RFZ5, D6RC05, D6RE74, D6RGE7, O95251, D6RBB3, Q9BVI0
GOTERM_CC_DIRECT	GO:0000123~histone acetyltransferase complex	3	0.69444444	0.08795964	D6RAS5, D6RFK0, D6RCS1, Q6IE81, D6RFZ5, D6RC05, D6RE74, D6RGE7, O95251, D6RBB3, Q9BVI0

Annotation Cluster 11	**Function: steroid/arachidonic acid metabolism (ES = 1.12)**				
Category	Term	Count	%	PValue	Genes
GAD_DISEASE	ulcer, gastric; repaglinide pharmacology; coagulation disorder	3	0.69444444	0.00326715	Q9UQ59, S5RV29, G3V188, S5RXA4, P33261, S5RG22, P10632, P11712, S5RV20, Q9UEH3, A0A087X125, S5R8H1, S5R8G8, E9PLI9, B7Z1F5, E9PIW6
GOTERM_BP_DIRECT	GO:0097267~omega-hydroxylase P450 pathway	3	0.69444444	0.01655131	Q9UQ59, S5RV29, G3V188, S5RXA4, P33261, S5RG22, P10632, P11712, S5RV20, Q9UEH3, A0A087X125, S5R8H1, S5R8G8, E9PLI9, B7Z1F5, E9PIW6
GAD_DISEASE	Epilepsy|	3	0.69444444	0.02229885	Q9UQ59, S5RV29, G3V188, S5RXA4, P33261, S5RG22, P10632, P11712, S5RV20, Q9UEH3, A0A087X125, S5R8H1, S5R8G8, E9PLI9, B7Z1F5, E9PIW6
GOTERM_BP_DIRECT	GO:0042738~exogenous drug catabolic process	3	0.69444444	0.02901813	Q9UQ59, S5RV29, G3V188, S5RXA4, P33261, S5RG22, P10632, P11712, S5RV20, Q9UEH3, A0A087X125, S5R8H1, S5R8G8, E9PLI9, B7Z1F5, E9PIW6
GAD_DISEASE	Myocardial Infarction|Stroke	3	0.69444444	0.06045948	Q9UQ59, S5RV29, G3V188, S5RXA4, P33261, S5RG22, P10632, P11712, S5RV20, Q9UEH3, A0A087X125, S5R8H1, S5R8G8, E9PLI9, B7Z1F5, E9PIW6
GAD_DISEASE	Drug Hypersensitivity	3	0.69444444	0.11803582	Q9UQ59, S5RV29, G3V188, S5RXA4, P33261, S5RG22, P10632, P11712, S5RV20, Q9UEH3, A0A087X125, S5R8H1, S5R8G8, E9PLI9, B7Z1F5, E9PIW6
GOTERM_BP_DIRECT	GO:0017144~drug metabolic process	3	0.69444444	0.12391974	Q9UQ59, S5RV29, G3V188, S5RXA4, P33261, S5RG22, P10632, P11712, S5RV20, Q9UEH3, A0A087X125, S5R8H1, S5R8G8, E9PLI9, B7Z1F5, E9PIW6
KEGG_PATHWAY	hsa00591:Linoleic acid metabolism	3	0.69444444	0.19034982	Q9UQ59, S5RV29, G3V188, S5RXA4, P33261, S5RG22, P10632, P11712, S5RV20, Q9UEH3, A0A087X125, S5R8H1, S5R8G8, E9PLI9, B7Z1F5, E9PIW6
GOTERM_BP_DIRECT	GO:0008202~steroid metabolic process	3	0.69444444	0.25418562	Q9UQ59, S5RV29, G3V188, S5RXA4, P33261, S5RG22, P10632, P11712, S5RV20, Q9UEH3, A0A087X125, S5R8H1, S5R8G8, E9PLI9, B7Z1F5, E9PIW6
KEGG_PATHWAY	hsa00590:Arachidonic acid metabolism	3	0.69444444	0.5059576	Q9UQ59, S5RV29, G3V188, S5RXA4, P33261, S5RG22, P10632, P11712, S5RV20, Q9UEH3, A0A087X125, S5R8H1, S5R8G8, E9PLI9, B7Z1F5, E9PIW6
KEGG_PATHWAY	hsa00982:Drug metabolism - cytochrome P450	3	0.69444444	0.56478116	Q9UQ59, S5RV29, G3V188, S5RXA4, P33261, S5RG22, P10632, P11712, S5RV20, Q9UEH3, A0A087X125, S5R8H1, S5R8G8, E9PLI9, B7Z1F5, E9PIW6

Annotation Cluster 12	**Function: innate immunity (ES = 0.97)**				
Category	Term	Count	%	PValue	Genes
UP_SEQ_FEATURE	Leucin Rich Repeats LRR 8	8	1.85185185	0.03153886	L8E7L4, B6UV79, B4DXB5, A0A0C4DH17, A0A0C4DFY3, A0A0A0MRE3, Q9Y4C4, G3V5X4, H3BLT9, Q8WXH0, H0YA04, Q6UXK5, T2HV29, Q14392, A0A087WZ24, Q7RTR2, P59046, A0A1D5RMN4, A0A1S0QI99, Q96RT1, C9JDA1, H0Y9E8, B4DIP2, P23945, A0A0C4DGK3, F8WBM4
UP_SEQ_FEATURE	Leucin Rich Repeats LRR 7	8	1.85185185	0.06797067	L8E7L4, B6UV79, B4DXB5, A0A0C4DH17, A0A0C4DFY3, A0A0A0MRE3, Q9Y4C4, G3V5X4, H3BLT9, Q8WXH0, H0YA04,

					Q6UXK5, T2HV29, Q14392, A0A087WZ24, Q7RTR2, P59046, A0A1D5RMN4, A0A1S0QI99, Q96RT1, C9JDA1, H0Y9E8, B4DIP2, P23945, A0A0C4DGK3, F8WBM4
UP_SEQ_FEATURE	Leucin Rich Repeats LRR 6	8	1.85185185	0.13395352	L8E7L4, B6UV79, B4DXB5, A0A0C4DH17, A0A0C4DFY3, A0A0A0MRE3, Q9Y4C4, G3V5X4, H3BLT9, Q8WXH0, H0YA04, Q6UXK5, T2HV29, Q14392, A0A087WZ24, Q7RTR2, P59046, A0A1D5RMN4, A0A1S0QI99, Q96RT1, C9JDA1, H0Y9E8, B4DIP2, P23945, A0A0C4DGK3, F8WBM4
UP_SEQ_FEATURE	Leucin Rich Repeats LRR 5	8	1.85185185	0.190516	L8E7L4, B6UV79, B4DXB5, A0A0C4DH17, A0A0C4DFY3, A0A0A0MRE3, Q9Y4C4, G3V5X4, H3BLT9, Q8WXH0, H0YA04, Q6UXK5, T2HV29, Q14392, A0A087WZ24, Q7RTR2, P59046, A0A1D5RMN4, A0A1S0QI99, Q96RT1, C9JDA1, H0Y9E8, B4DIP2, P23945, A0A0C4DGK3, F8WBM4
UP_SEQ_FEATURE	Leucin Rich Repeats LRR 4	8	1.85185185	0.26123635	L8E7L4, B6UV79, B4DXB5, A0A0C4DH17, A0A0C4DFY3, A0A0A0MRE3, Q9Y4C4, G3V5X4, H3BLT9, Q8WXH0, H0YA04, Q6UXK5, T2HV29, Q14392, A0A087WZ24, Q7RTR2, P59046, A0A1D5RMN4, A0A1S0QI99, Q96RT1, C9JDA1, H0Y9E8, B4DIP2, P23945, A0A0C4DGK3, F8WBM4

Annotation Cluster 13	**Function: Blood coagulation (ES = 0.94)**				
Category	Term	Count	%	PValue	Genes
UP_SEQ_FEATURE	domain:F5/8 type C	3	0.69444444	0.03620894	Q9UHC6, Q5T241, H0Y570, A0A087WTA1, Q16832, Q5T244, E9PDN6, Q5T245, Q9C0A0, A0A024R906, F5H107, A0A0A0MR20
SMART	SM00231:FA58C	3	0.69444444	0.08352304	Q9UHC6, Q5T241, H0Y570, A0A087WTA1, Q16832, Q5T244, E9PDN6, Q5T245, Q9C0A0, A0A024R906, F5H107, A0A0A0MR20
INTERPRO	IPR000421:Coagulation factor 5/8 C-terminal type domain	3	0.69444444	0.1055893	Q9UHC6, Q5T241, H0Y570, A0A087WTA1, Q16832, Q5T244, E9PDN6, Q5T245, Q9C0A0, A0A024R906, F5H107, A0A0A0MR20
INTERPRO	IPR008979:Galactose-binding domain-like	3	0.69444444	0.54643549	Q9UHC6, Q5T241, H0Y570, A0A087WTA1, Q16832, Q5T244, E9PDN6, Q5T245, Q9C0A0, A0A024R906, F5H107, A0A0A0MR20

Annotation Cluster 14	**Function: subcellular (cytoskeleton) localization (ES = 0.81)**				
Category	Term	Count	%	PValue	Genes
GOTERM_CC_DIRECT	GO:0035253~ciliary rootlet	3	0.69444444	0.01879502	P33176, Q12840, O60282
UP_SEQ_FEATURE	region of interest:Globular	3	0.69444444	0.04083697	P33176, Q12840, O60282
GOTERM_MF_DIRECT	GO:0008574~ATP-dependent microtubule motor activity, plus-end-directed	3	0.69444444	0.05527477	P33176, Q12840, O60282
GOTERM_BP_DIRECT	GO:0030705~cytoskeleton-dependent intracellular transport	3	0.69444444	0.06156746	P33176, Q12840, O60282

UP_SEQ_FEATURE	domain:Kinesin-motor	3	0.69444444	0.224336	P33176, Q12840, O60282
INTERPRO	IPR019821:Kinesin, motor region, conserved site	3	0.69444444	0.23080968	P33176, Q12840, O60282
INTERPRO	IPR001752:Kinesin, motor domain	3	0.69444444	0.26383411	P33176, Q12840, O60282
SMART	SM00129:KISc	3	0.69444444	0.27840794	P33176, Q12840, O60282
GOTERM_CC_DIRECT	GO:0005871~kinesin complex	3	0.69444444	0.31943138	P33176, Q12840, O60282
GOTERM_MF_DIRECT	GO:0003777~microtubule motor activity	3	0.69444444	0.5421099	P33176, Q12840, O60282
UP_KEYWORDS	Motor protein	3	0.69444444	0.76065031	P33176, Q12840, O60282

Source = identify the source of functional terms associated; UP_SF = UP_SEQ_FEATURE; UP_K = UP_KEYWORD; IP = INTERPRO; GO_DIR = GOTERM_MF_DIRECT; GO_BP = GOTERM_BP_DIRECT; GO_CC = GOTERM_CC_DIRECT; KEGG = KEGG_PATHWAY; COG_ONT = COG_ONTOLOGY. N = number of Uniprot associated genes. The red written identifies non-significant (P > 0.05) gene ontology Terms.
